# Mathematical modeling of disinformation and effectiveness of mitigation policies

**DOI:** 10.1038/s41598-023-45710-2

**Published:** 2023-10-31

**Authors:** David J. Butts, Sam A. Bollman, Michael S. Murillo

**Affiliations:** Department of Computational Mathematics, Science and Engineering, Michigan State University, East Lansing, 48824 USA

**Keywords:** Human behaviour, Computational science, Applied mathematics

## Abstract

Disinformation is spread to manipulate public opinion for malicious purposes. Mathematical modeling was used to examine and optimize several strategies for combating disinformation—content moderation, education, and counter-campaigns. We implemented these strategies in a modified binary agreement model and investigated their impacts on properties of the tipping point. Social interactions were described by weighted, directed, and heterogeneous networks. Real social network data was examined as well. We find that content moderation achieved by removing randomly selected agents who spread disinformation is comparable to that achieved by removing highly influential agents; removing disinformation anywhere in a network could be an effective way to counter disinformation. An education strategy that increases public skepticism was more effective than one that targets already biased agents. Successful counter-campaign strategies required a substantial population of agents to influence other agents to oppose disinformation. These results can be used to inform choices of effective strategies for combating disinformation.

## Introduction

The spread of disinformation has brought numerous adverse consequences, such as the manipulation of the 2016 US presidential election^1,2^, COVID vaccine hesitancy^3–5^, and the growth of QAnon^6^. Advances in the development of chatbots are creating new concerns^7–10^. In response, considerable efforts have been devoted to detecting^11–14^ and combating^15–18^ disinformation. Disinformation tracking, bot detection, and credibility scoring tools^19^ have been developed, but the spread of malicious information remains a major challenge. Disinformation has escalated to a degree that the US Congress is examining intervention policies^20^. These policies can be divided into two categories: individual-empowering and structure-changing policies^21^.

Individual-empowering policies help individuals to evaluate information they are exposed to and include policies such as fact-checking social-media platforms^18^. Ideally, when social-media users interact with disinformation, they would be warned and discouraged from believing or spreading it further^21^. The reliability of information sources can also be rated^22^. This rating can be performed by experts or users who either rate many articles from a single source, generating an aggregate score, or rate sources directly. Individuals are more skeptical of sources with low ratings and are less likely to interact with information from such sources^22^. In addition to fact-checking and rating sources, one of the most powerful ways to empower individuals is through education. For example, instructional materials for teaching critical thinking can be created, such as guides for librarians to teach students to be aware of fake news^17^, or for teachers to teach young students to think critically about news they come across on social media^15^.

In contrast to individual-empowering policies, structure-changing policies prevent individuals from being exposed to certain disinformation entirely. Policies that fall in this category are primarily implemented by social-network operators who monitor the content on their sites and remove users or content they deem unacceptable^20^. Additionally, groups can run counter-campaigns on social media to drown out disinformation with facts^18,23,24^.

Mathematical models are often used to evaluate, choose and optimize strategies for implementing such policies for combating disinformation, because it is usually impractical to test such strategies in the real world. Many models can be applied to study the spread of disinformation^25,26^, including the voter model^27–29^, the Axelrod model^30^, epidemiological models^31–35^, the attraction-repulsion model^36^, the naming-game model^37,38^, and the binary agreement model^39–43^.

In this work, we adopt a policy-driven approach to understanding and combating disinformation, diverging from the prevalent trend in the literature that often emphasizes the mathematical or statistical mechanics of models. By both offering the theoretical underpinnings of our modeling approach and linking our modeling directly to real-world policies, we hope to provide insights that can aid in effective policymaking.

Towards this goal, we employ the binary agreement model with committed minorities, an agent-based model developed by Xie et al. This model was chosen not merely because it is well studied but because it encapsulates critical properties of disinformation spread. Furthermore, it highlights a pivotal moment in majority opinion, influenced by a committed minority that permits minority rule. In the binary agreement model, a tipping point occurs when a critical fraction of a network population, $p_c$, which is only approximately one tenth of the population on complete graphs, advocates a particular opinion strongly^39^. On heterogeneous graphs, $p_c$ is even lower, as the average connectivity of the graph decreases^44^; such low values of $p_c$ make it very challenging to mitigate disinformation. The fact that this model exhibits a tipping point is important because tipping points have been observed in real human interactions. For example, in groups assigned to identify an item in an image, a 25% minority can sway the majority’s answer^41^. Moreover, a goal of disinformation campaigns is often to sway public opinion towards a tipping point. The ability of a committed minority to overtake majority opinions can have large effects in the real world, as has been seen with social-media influence campaigns leading up to the 2016 US presidential election^1,2^, influence campaigns that have affected societal responses to the COVID-19 pandemic^3–5^, and the rise of QAnon^6^.

The basic binary agreement model of Xie et al.^39^ can be extended to incorporate more realistic features. Examples of extensions that have been developed include modifying the propensity of an agent in the mixed state to share one of its opinions^45^, varying the number of interactions needed for an agent to change opinions^45,46^, adding a competing committed minority^40^, expanding the number of possible opinions^47^, varying the level of commitment of the minority^38^, and including heterogeneous^48^ and dynamic^49^ graphs.

While many studies have examined the effects of a single change to the binary agreement model on the dynamics of the model, to our knowledge, no study has examined the effects of incorporating one or more disinformation-management strategies. Here, we explore strategies that have previously been identified as potential real-world strategies for combating the spread of disinformation^20^. We implement individual-empowering and structure-changing policies—in the form of content-moderation, education, and counter-campaign strategies—in a modified version of the binary agreement model, and we examine their ability to move, smooth, or remove the tipping point exhibited by the model when implemented on several weighted, heterogeneous networks. We incorporate these strategies into our model by removing or adding agents or altering agents’ susceptibility to other opinions. Properties of the tipping point provide us with metrics that can be used to quantify the effectiveness of various strategies for countering disinformation. We apply our methods to several types of synthetic networks, including small-world and scale-free networks, that capture many features of social networks. Additionally, we apply our methods to real social-network data, from Asian users of LastFM^50^.

## Methods

### Binary agreement model and its computational implementation

We construct agent-based models in which agents are connected by a graph and follow the binary agreement model proposed by Xie et al.^39^. Each agent can hold one of the single opinions *A* or *B*, or the mixed opinion *AB*, and can be either committed or uncommitted to that opinion. Pairs of agents update their opinions through one of 12 interactions, using the update rules given by Xie et al.^39^; for convenience, these update rules are summarized in Table 1. We refer to these basic update rules as the opinion update rules.

**Table 1:** Binary agreement model update rules for the three opinions A, B and AB. Here, our convention is that opinion A is disinformation.

Before interaction (${\text {speaker}} \xrightarrow {\text {opinion}} \text {listener}$)	After interaction (speaker–listener)
$A\xrightarrow {A}A$	$A-A$
$A\xrightarrow {A}B$	$A-AB$
$A\xrightarrow {A}AB$	$A-A$
$B\xrightarrow {B}A$	$B-AB$
$B\xrightarrow {B}B$	$B-B$
$B\xrightarrow {B}AB$	$B-B$
$AB\xrightarrow {A}A$	$A-A$
$AB\xrightarrow {A}B$	$AB-AB$
$AB\xrightarrow {A}AB$	$A-A$
$AB\xrightarrow {B}A$	$AB-AB$
$AB\xrightarrow {B}B$	$B-B$
$AB\xrightarrow {B}AB$	$B-B$

Each row of the table shows one possible interaction. In the left column, the speaker’s state is listed at the tail of the arrow, the opinion that speaker shares is above the arrow, and the listener’s state is at the head of the arrow. The right column shows the outcome of the interaction; the speaker’s state is listed first followed by the listener’s state.

Committed agents are introduced by choosing a fraction, $p_a$, of agents who always hold the opinion *A*, regardless of their interactions with others. In our model, this committed minority spreads disinformation; i.e., the opinion *A* is disinformation, and the opinion *B* is the truth. In general, $p_a$ is varied over a wide range to find the critical value that defines the model’s tipping point; disinformation mitigation strategies aim to move this tipping point to a more favorable value.

**Figure 1:**
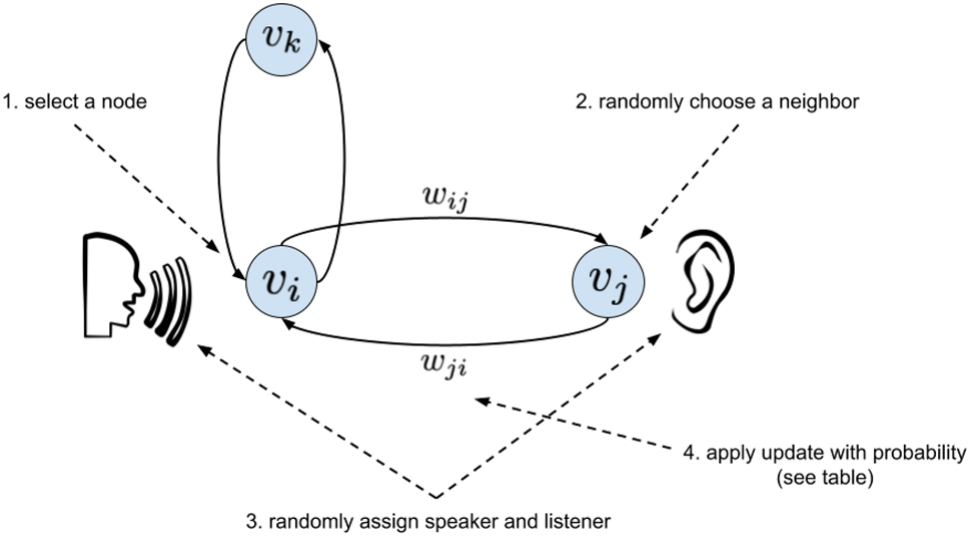
Simulation update rules governing computational implementation of the binary agreement model. At each time step, for each node $v_i$ (i.e., each individual) in the graph (step 1), one of its neighbors $v_j$ was selected randomly (step 2). In each pair of nodes, one was randomly assigned to be the speaker, and the other was assigned to be the listener (step 3). With probability $w_{ij}$ (the weight of the edge between the speaker and the listener), the nodes interacted and updated their opinions (step 4). See Table 1 for a table of opinion update rules.

We applied the opinion update rules to agents who were connected on simulated social networks, which will be discussed in the subsection below. At each time step, for each agent in the network, one of that agent’s neighbors was selected randomly and uniformly. For each such pair of agents, either the agent or the neighbor was randomly assigned to be the speaker, and the other was assigned to be the listener. Probabilistically, the speakers shared their opinions with the listeners; such interactions determined the new opinions of both the speakers and the listeners. We show these rules, which govern our computational implementation of our model, in Fig. 1. We refer to these rules as the simulation update rules.

Unless otherwise specified, all agents not committed to *A*, i.e., not in the committed minority, were initialized as uncommitted to *B*, i.e., as holding the opinion *B* but not committed to that opinion. The simulation was updated repeatedly, over many time steps, until either no agent changed state and none were in a mixed state, or 5000 time steps had passed. The fractional densities $n_A$ and $n_B$ of the nodes was then computed.

We examined the tipping point in our simulations by comparing the fraction of agents with opinion *B* (the truth), denoted by $n_B$, with the fraction of agents committed to the opinion *A* (disinformation), denoted by $p_a$. Many simulations on each graph type were performed for each anti-disinformation strategy and the basic binary agreement model; see the Simulations subsection below for further details. At the end of each simulation, we recorded the values of $n_B$ and $p_a$. We compared these results across simulations; for each strategy and the basic model, and for each graph type, we plotted $n_B$ vs. $p_a$ on a single plot. For the basic binary agreement model without any intervention, a tipping point is evident on the plot; at the tipping point, $n_B$ falls from approximately one to zero once $p_a\approx 0.1$, as is shown by the black dot-dash line in Figs. 4 and 5. We compare the locations of the tipping point, i.e., the value of $p_a$ at which $n_B$ begins to fall steeply toward zero, for each strategy to the location of the tipping point in the basic model and to the locations of the tipping points seen with other strategies.

The goal of our work was to measure the effects of several disinformation-mitigation strategies on the spread of disinformation using our model. To measure the spread of disinformation in our simulations, we examined to what extent each strategy moved, smoothed, or removed the tipping point exhibited by our model. Concretely, to measure how much a strategy moved the tipping point relative to another condition, we measured relative changes in the value of $p_a$ at the tipping point between conditions. Smoothing and removal of a tipping point were noted as qualitative changes. These are the outputs of our simulations that we examine in the Results section below.

### Simulated social networks

Artificial social networks were modeled using weighted, directed graphs $G=(V,E)$. Agents $v_i\in V$ were represented by the graph’s vertices, and an edge between agents, $(v_i,v_j,w_{ij})\in E$, represented a connection that allowed $v_i$ to interact with $v_j$ with probability $w_{ij}$. We generated several graph types that were initially undirected and unweighted, all of which were created using NetworkX^51^. The graphs we generated, and their required arguments, are listed in Table 2. We created random and small-world networks by varying the probability of creating an edge in Erdős-Rényi graphs, and by varying the initial number of edges and the probability of rewiring an edge in Watts–Strogatz graphs. Scale-free graphs were created by tuning the number of edges to attach from a new node to existing nodes in Barabási-Albert graphs. Additionally, we examined barbell, lattice, and complete graphs.

**Table 2:** Graphs that were explored and their parameters.

Graph type	N	k	p	m	n	${m_1}$	${m_2}$
complete_graph	400						
watts_strogatz_graph (small world)	400	0.02*N*	.5				
watts_strogatz_graph (small world)	400	0.12*N*	.5				
watts_strogatz_graph (small world)	400	0.25*N*	.5				
watts_strogatz_graph (random)	400	0.02*N*	1				
watts_strogatz_graph (random)	400	0.12*N*	1				
watts_strogatz_graph (random)	400	0.25*N*	1				
erdos_renyi_graph (random)	400		0.02				
erdos_renyi_graph (random)	400		0.12				
erdos_renyi_graph (random)	400		0.25				
barabasi_albert_graph	400			4			
barabasi_albert_graph	400			24			
barabasi_albert_graph	400			50			
grid_2d_graph				200	200		
barbell_graph						199	2

We list the graph types and their required arguments. For the Watts–Strogatz graphs, the argument *k* is the initial number of nearest neighbors that are connected, and *p* is the probability of rewiring an edge. For the Erdős-Rényi graphs, *p* is the probability of creating an edge between a pair of nodes. For the grid graph, the arguments *m* and *n* are the dimensions of the grid. For the barbell graph, $m_1$ is the number of nodes in each of two fully connected subgraphs that are attached by $m_2$ intermediate nodes. Every graph had 400 nodes, but graphs had different numbers of edges. We also explored a real-world graph of Asian users of LastFM^50^, not listed here.

**Figure 2:**
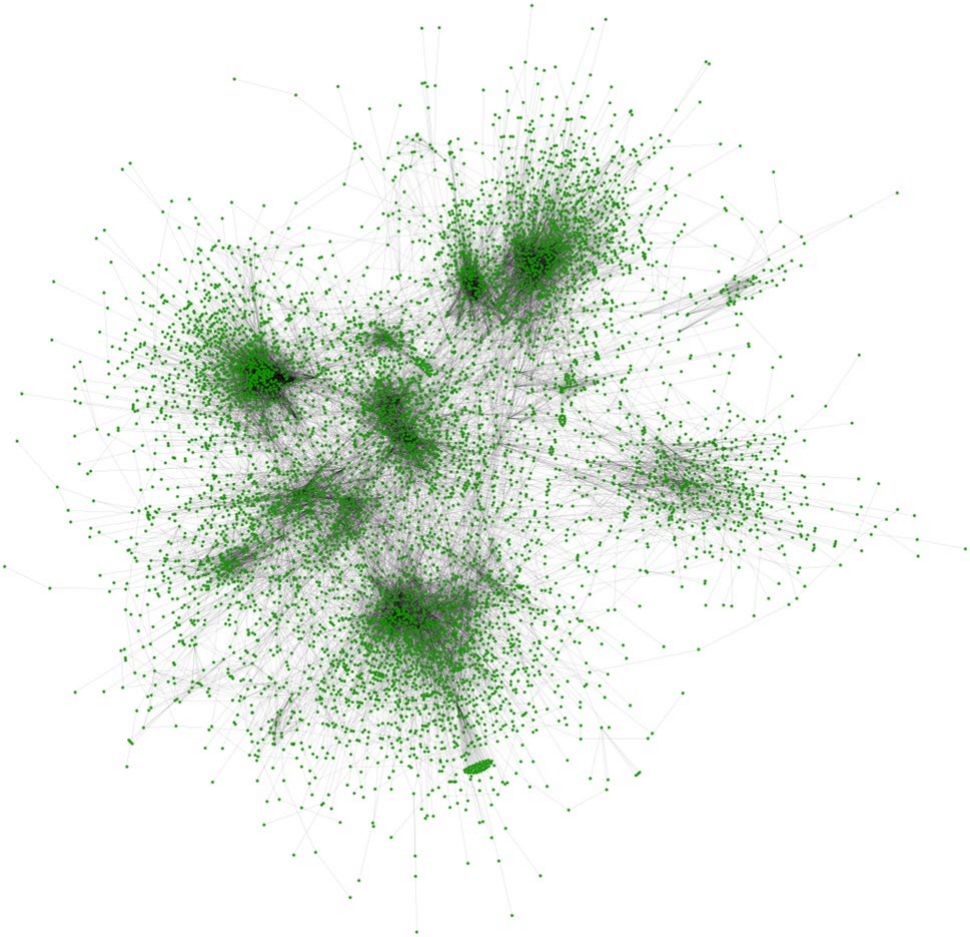
Asian users of the LastFM social network. Users are represented as green nodes, and edges represent a mutual follower relationship between users. This social network consists of 7624 nodes and 27,806 edges. The placements of the nodes were determined using the Yifan Hu algorithm^52^.

Real social networks can be very large. Here, our numerical results are based on relatively small graphs with 400 nodes, a value chosen to minimize computational cost while retaining accuracy. This value was chosen by executing the binary agreement model on networks of progressively larger size and comparing the steady state of the simulations to the mean-field (large-node) limit of the model. Consistent with Xie et al. (see Fig. 1a)^39^, we found that results obtained with a complete graph with 400 nodes agree well with the mean-field approximation. However, as a final study, we performed simulations on the real social network of Asian users of LastFM^50^, which has 7624 nodes and 27,806 edges; this result will be discussed in the next section. A graph of the Asian users of LastFM is shown in Fig. 2.

The basic topology of *G* contains the presence or absence of an edge. Each edge was converted into two weighted, directed edges that formed a loop between the two connected nodes. These edges can be can be assigned real-valued edge weights that reflect the relative strength of a social interaction. We used a graph’s structure to determine its weights; nodes were more likely to interact with neighbors that they shared more connections in common with. Similar to graph transitivity, we define social transitivity as the ratio of the number of neighbors shared between the successor and predecessor nodes of an edge to the total number of neighbors of the predecessor node. We then weighted each edge by its social transitivity. Mathematically, the weights in the graph are given by

1 $$\begin{aligned} w_{ij} = \frac{1 + \text {number of shared neighbors between }v_i \text { and }v_j}{\text {number of neighbors of }v_i}. \end{aligned}$$

Here, the additional 1 in the numerator accounts for the fact that $v_i$ and $v_j$ share a connection. We used these weights to encode the probability that two nodes will interact in our model. A listener that shares many neighbors with a speaker has a higher probability of switching state than one that shares few neighbors with the speaker.

### Mitigation strategies

Using our modified version of the binary agreement model, we evaluated three mitigation policies—content moderation, education, and counter-campaigns—that are discussed in the following subsubsections. In this subsection, we first provide an overview of the strategies we consider for implementing those policies, and the implementation of these strategies in our model. In the subsubsections below, we discuss details of their implementation in our model.

We first discuss content moderation, in the form of banning some users who spread disinformation from social media platforms. We implemented two content-moderation strategies in our model by removing agents from our graphs in two ways: removing influential agents in the committed minority that spreads disinformation, or removing randomly chosen agents in the committed minority.

Next, we consider two educational strategies. These strategies aim to educate individuals broadly, and target those who are interacting with disinformation. Broad education consists of teaching individuals how to identify disinformation to reduce the chances that they will believe disinformation or hold immutable opinions. We refer to this strategy as a skepticism strategy, and we implemented it in our model by reducing committed agents’ level of commitment to ideas. In contrast, a targeted educational strategy includes fact-checking information and labeling sources, for example by providing labels on online videos or warnings on cigarettes, to bias individuals towards the truth. We refer to this strategy as an attentive strategy, and implemented it in our model by giving all non-committed agents a bias towards sharing and believing the truth.

Finally, we discuss counter-campaign strategies that counter disinformation with facts. A counter-campaign strategy can be driven by large agencies; for example, the US Centers for Disease Control and Prevention provides guidance about sharing vaccine information^53^. Alternatively, counter-campaigns can be conducted by groups of people who share information to combat disinformation directly. We modeled counter-campaign strategies by introducing a second committed minority that is committed to the truth and competes with the original committed minority that is spreading disinformation.

**Figure 3:**
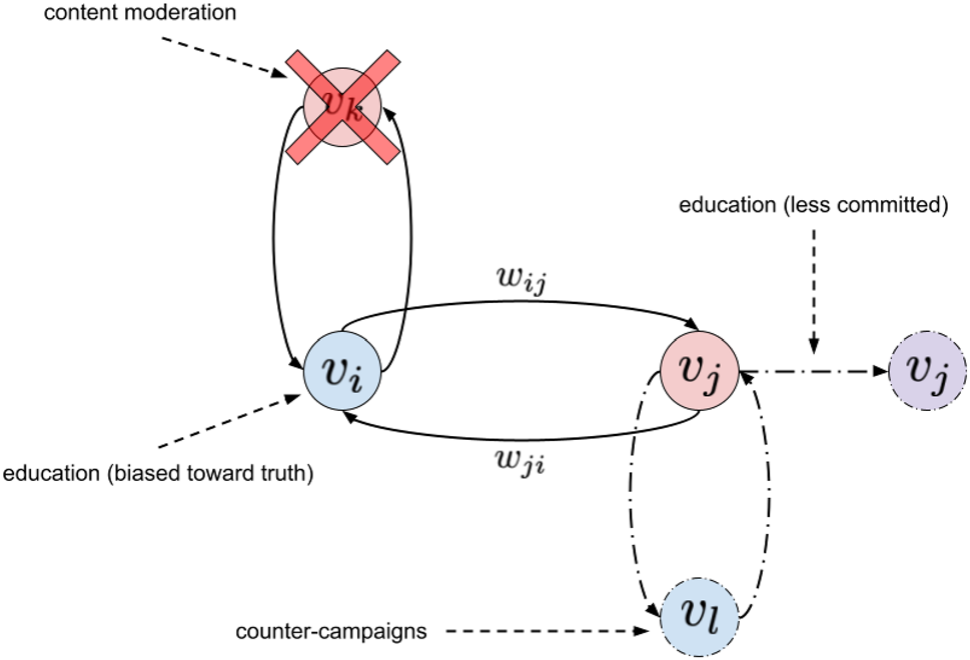
Mitigation strategies examined using our model. Solid arrows represent edges in the graph, dot-dashed arrows represent a change made to the graph structure, and dashed arrows represent comments. Blue nodes represent agents with the true opinion, red nodes represent agents in the committed minority who believe disinformation, and purple nodes represent agents with variable commitment to an opinion. Two strategies for content moderation, both involving banning users from a network, were implemented in our model by removing disinformation-spreading, committed-minority nodes from a graph. An education strategy aimed at increasing skepticism was implemented by reducing the level of commitment to ideas of committed-minority nodes. Another education strategy that brought attention to disinformation was implemented by biasing all non-committed nodes towards the truth. We also introduced counter-campaigns into a graph by adding a second committed minority that was committed to the truth. Each strategy was investigated separately.

Using our model, we examined the effects of each of these strategies separately. The strategies we implemented in our model are shown in Fig. 3, and the policies, specific strategies, and our implementations of them in our model are given in Table 3.

**Table 3:** Policies and strategies for combating disinformation and their implementations in our model.

Policy	Strategy	Implementation in model	Result
Content moderation	Temporary or permanent bans of highly connected users spreading disinformation Temporary or permanent bans of randomly chosen users spreading disinformation	Removing influential agents in the committed minority, i.e., influential committed-minority nodes in a graph Removing randomly chosen agents in the committed minority, i.e., randomly chosen committed-minority nodes in a graph	Slightly increases required size of committed minority for tipping point Random and targeted approach perform similarly, except on graphs with unique topology, e.g., barbell
Education	Skepticism: teaching critical thinking Attention: providing individuals with ratings for sources and fact-checking of information	Reducing the level of commitment to ideas of agents, i.e., nodes, in the committed minority Giving all agents, i.e., nodes, not in the committed minority a bias towards towards the truth	Skepticism greatly increases the required size of a committed minority Attention has little to no effect on the tipping point
Counter-campaigns	Spread of facts by groups to counter disinformation	Introducing an opposing minority of agents, i.e., nodes, committed to the truth	Larger counter-campaigns increase the tipping value of $p_a$

The policies we considered are given in the first column. In the middle column, we list the specific strategies for implementing these policies that we examined in this work. In the third column, we list our implementations of these strategies in our model. In the final column, we summarize the effect each policy has on the tipping point in the binary agreement model.

#### Content moderation

We examined the strength of content moderation by removing different proportions of committed agents from a graph over an order-of-magnitude range; we removed 0.25%, 0.5%, 1%, 2%, or 2.5% of the total number of nodes in the graph, for values of $p_a$ that varied from 0.03 to 0.13. Committed nodes were removed before we executed each simulation examining content-moderation strategies.

One content-moderation strategy we considered required removing influential agents in the committed minority that were spreading disinformation. Thus, we needed to identify influential nodes in the committed minority in a graph. We define influence using two key metrics: degree centrality and betweenness centrality. Degree centrality measures the number of connections an agent has, while betweenness centrality measures how centrally located an agent is in the graph. Both measures of centrality are normalized to fall between zero and one. The degree centrality of a node is how many connections that node has divided by the total number of possible connections, while its betweenness centrality is calculated as how many shortest paths pass through that node divided by the total number of shortest paths in the graph. An agent with both high degree centrality and high betweenness centrality has the potential to spread disinformation directly to many agents and to spread disinformation to disparate parts of a graph that might not interact without that agent. Using these metrics and a normalization method, we define the influence of a node as:

2 $$\begin{aligned} {{{\mathscr {I}}}}(v) = \frac{1}{2}\bigg (C_D(v) + C_B(v)\bigg ), \end{aligned}$$

where $C_D$ is the degree centrality and $C_B$ is the betweenness centrality. This metric allowed us to identify influential nodes in the committed minority. In each simulation in which influential committed-minority nodes were removed, the most influential committed-minority nodes were removed, up to the desired percentage of all nodes in the graph.

Clearly, if enough nodes were removed, the size of a committed minority would be too small for it to overtake the majority. Moreover, the size of the committed minority could be decreased by removing nodes at random as well as by removing influential nodes. Therefore, we compared the effects of our strategy of removing the most influential committed-minority nodes against the effects of a second strategy of removing randomly selected committed-minority nodes.

#### Education

Another mitigation policy we examined was education. In particular, we examined two strategies based on education: skepticism and attention. We implement a skepticism strategy in our model by relaxing the strength of commitment, c, held by the committed minority and endowing committed agents with a probability of changing their opinion after an interaction. This probability is defined as

3 $$\begin{aligned} p(c) = {\left\{ \begin{array}{ll} 2(c-c_{min})/(1-c_{min})^2 &{} c_{min}\le c\le 1, \\ 0 &{} {\text {else}}. \\ \end{array}\right. } \end{aligned}$$

Here, $c_{min}$ is the lowest level of commitment that a committed individual can have; we assume $c_{min}=1/2$. Equation 3 is a triangular distribution that linearly increases between zero and four when $1/2\le c\le 1$. Note that as $c_{min}$ tends to one, *p*(*c*) tends towards a delta function centered at $c=1$. This implies that all agents are fully committed, and thus we recover the original binary agreement model. In this strategy, a partially committed agent becomes fully uncommitted if it switches its opinion to the mixed state.

We implemented an attention strategy consisting of fact checking and source rating into our model by biasing non-committed agents towards the truth. In our model, the true opinion is *B*, and we assume that our attention strategy leads agents to be more likely to believe *B*. Therefore, agents in the mixed state are more likely to switch their opinion to *B* than to *A*. Additionally, those who already believe the truth have a preference toward keeping that opinion. To implement these two effects, we introduce a truth bias, $\beta =0.1$, that is the difference between the probability that an agent with the mixed opinion will share *B* in an interaction with a neighbor and the probability that the agent will share *A* in an interaction. The truth bias $\beta$ is also used to increase the probability that an agent with opinion *B* will change its opinion after an interaction. Mathematically, we have $P(AB{\text { shares }}B)-P(AB{\text { shares }}A)=\beta$, and $P(B{\text { switches to }}AB|{\text {neighbor shared }}A) = 1-\beta$.

#### Counter-campaigns

Counter-campaigns were implemented by initializing a group of agents to be committed to the opinion *B*, which is the truth, to combat disinformation from those who are committed to *A*, which is disinformation. To examine the effects of different sizes of counter-campaigns, we considered competing minorities consisting of either $5\%$ or $15\%$ of the total population, and we initialized these minorities to be committed to *B*. We assumed that counter-campaigns would begin in a local region of the graph, similar to an echo-chamber. This effect was incorporated by initializing the graphs in a non-random way, without changing the topology of the graph. Using NetworkX’s “spring_layout” routine that implements the Fruchterman-Reingold force-directed algorithm, we assigned a position to each node in the graph that was contained in the box $[-1,-1]\times [1,1]$. We then ensured that all nodes that were committed to *A* were initialized on the left side of the graph (their first coordinate was negative), and that those that were committed to *B* were on the right side (their first coordinate was positive). All remaining individuals were initialized as uncommitted to *B*, i.e., holding the opinion *B* but not committed to that opinion.

### Simulations

For each of the six anti-disinformation strategies, we conducted 30 simulations on a new instantiation of each of the 15 graph topologies listed in Table 2 and 30 simulations on the real social network. Each of these 2880 simulations executed our model either 11 (content moderation) or 15 (education and counter campaigns) times, corresponding to the values of $p_a$ we examined, and had a new initial configuration of committed agents. In total, our model was executed 118,080 times, and required over 18,000 core hours on a distributed memory supercomputer. Each simulation was run until either no agent changed state and none were in a mixed state, or 5000 time steps had passed. The results of these simulations were averaged for each graph topology; this allowed us to examine the variance in outcomes and outlier results.

## Results

We investigated the effects of three anti-disinformation policies, implemented in the form of six specific strategies for combating disinformation, using a binary agreement model we modified to incorporate these strategies. We conducted a total of 2880 simulations on 15 different graph topologies to explore the effects of each of these strategies, and an additional 30 simulations on a real social network. At the end of each simulation, we recorded the fraction of agents with opinion *B* at steady state and the fraction of agents committed to the opinion *A*. Following the notation in Xie et al.^40^, these values are denoted by $n_B$ and $p_a$, respectively. For each anti-disinformation strategy, we plotted $n_B$ vs. $p_a$ and examined how the value of $p_a$ at the tipping point varied among the strategies employed. We also examined whether a strategy affected the shape of the tipping point or removed the tipping point altogether. Below, we discuss our results for each strategy. Because of space limitations, we show results from complete graphs and the real social network in the main manuscript; the results for all graph types are shown in the Supplement.

### Content moderation

**Figure 4:**
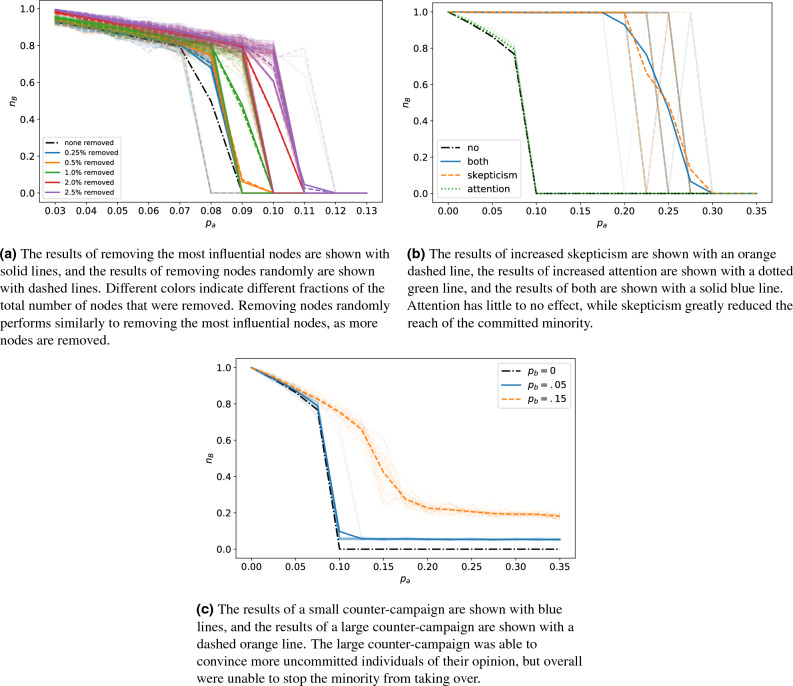
Strategies based on **a** content moderation, **b** education, and **c** counter-campaigns applied to a complete graph. In each subfigure, the fraction of nodes with opinion *B* at steady-state, $n_B$, is plotted versus the fraction of nodes committed to *A*, $p_a$. For reference, the black dot-dash line shows how $n_B$ varies with $p_A$ when no nodes are removed, i.e., for the basic binary agreement model in the absence of a mitigation strategy. Each opaque line shows an average of 30 simulations, while the partially transparent lines represent individual simulations.

**Figure 5:**
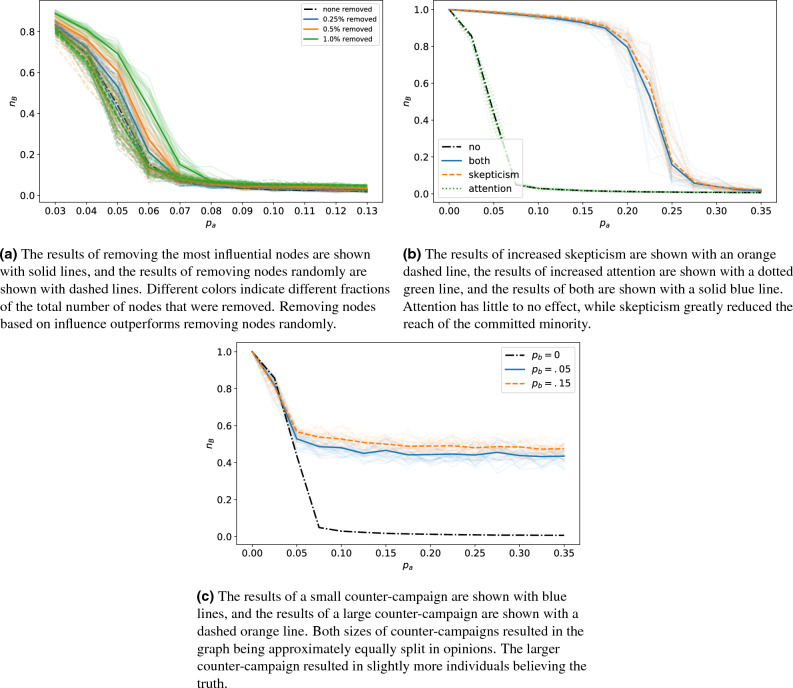
Strategies based on **a** content moderation, **b** education, and **c** counter-campaigns applied to the Asian users of the LastFM network^50^. In each subfigure, the fraction of nodes with opinion *B* at steady-state, $n_B$, is plotted versus the fraction of nodes committed to *A*, $p_a$. For reference, the black dot-dash line shows how $n_B$ varies with $p_A$ when no nodes are removed, i.e., for the basic binary agreement model in the absence of a mitigation strategy. Each opaque line shows the average of 30 simulations, while the partially transparent lines represent individual simulations.

Content moderation was implemented by removing committed nodes from a graph before we executed our simulations. Committed nodes were removed either based on their influence or randomly. In Fig. 4a, we show an example of the effects of content moderation on a complete graph, and in Fig. 5a we show the results on a real social network. Due to computational limitations, only up to 1% of the committed agents were removed from the real social network. Results for removing highly influential committed nodes are shown with solid lines, and results for removing committed nodes randomly are shown with dashed lines. Results for the basic binary agreement model in the absence of intervention are indicated with a black dot-dash line. Different line colors indicate different percentages of the total nodes that were removed. Each opaque line shows the average result of 30 simulations, and each partially transparent line shows the result of a single simulation. Similar plots for content moderation implemented on all of our graphs are shown in the Supplement.

As expected, as more nodes were removed from a graph, the initial size of the committed minority needed to be larger to overtake the majority; this feature was shared among all graph types. For most graphs, removing nodes randomly performed similarly, if not identically, to removing based on influence. However, on the real network, removing based on our influence metric outperformed removing randomly. As shown in Fig. 2, the LastFM social network has many clusters of nodes. These clusters are interconnected by a few nodes, and our metric does well at identifying these nodes. For barbell, grid, and the small world graphs with a low average degree, removing nodes randomly outperformed using our influence metric. The unique topology of the grid and barbell network ranks many of the nodes as equally important (e.g., nodes in the fully connected components of a barbell graph) and continuously ranks certain nodes as most important (e.g., the center node of the grid graph), while removing randomly we might find a better set of nodes. Once a large number of nodes have been removed, either method for removing nodes work equally well, except for our real network. These results suggest using the influence metric to remove a small number of nodes from a graph, unless the graph has special topology that may severely constrain how nodes are selected. If a large number of nodes (relative to the size of the graph) are being removed, a random approach is better due to the extra computational cost for little to no gain in performance.

### Education

We also examined the use of education that aimed to increase individual’s skepticism and attention. Individuals who are more skeptical of information are less likely to be fully committed to an opinion. Whereas attentive individuals are biased towards the truth. Here, we implemented skepticism by relaxing the commitment of individuals and attention by introducing a bias towards sharing and holding the opinion *B*. Using a complete graph in Fig. 4b and a real social network in Fig. 5b, we show the effects of skepticism with an orange dashed line, attention with a green dotted line, a combination of both with a solid blue line, and no education with a black dot-dashed line. Each opaque line is the average of 30 runs, and transparent lines are singular runs. We show similar plots for applying education policies to all graph types in the Supplement.

Attention had a small effect for all graph types. In random graphs, when an attention strategy was used the size of the committed minority needed to overtake the majority grew slightly with the average connectivity. Attention had no effect for on the real social network nor complete, barbell, and grid graphs. Contrary to attention, skepticism required the size of the committed minority to be between 30% and 35% in order to overtake the majority. Using both attention and skepticism together produces similar results as just using a strategy based on skepticism. These results suggest that educating people to be more skeptical can greatly reduce the dangers of a committed minority.

### Counter-campaigns

Additionally, we examined counter-campaigns that were implemented by introducing an additional group committed to the opinion *B*, denoted by $p_b$. For a small counter-campaign, we let $p_b=0.05$, and $p_b =0.15$ for a large one. In Fig. 4c, we examine the effects of different sized counter-campaigns on a complete graph, and a real social network in Fig. 5c. As before, we show the result of no intervention with a black dot-dashed line. We show the effects of the small counter-campaign with a solid blue line and the large counter-campaign with a dashed orange line. Each opaque line is the average of 30 simulations, and transparent ones are individual runs. Again, similar plots for counter-campaigns applied to all graph types are shown in the Supplement.

As expected, when a larger counter-campaign is used there is a larger portion of individuals with the opinion *B* at steady-state. The only exceptions are the barbell, grid, and real social network graphs, where small and large counter-campaigns have the same effect; this is likely due to their unique topologies. On most graphs when a small counter-campaign is introduced, the tipping point is located when $p_a$ is between 0.1 and 0.12, and past the tipping point $n_B \approx 0.05$: the size of the counter-campaign. This behavior is not observed on the grid graph, instead $n_B$ appears to decay exponentially to 0.4.

For most graphs, when a large counter-campaign is introduced the tipping point is located when $p_a$ is between 0.15 and 0.2, and past the tipping point $n_B \approx 0.18-0.3$. Again, the exceptions are the barbell, grid and real social network graphs, where large and small counter-campaigns act similarly and $n_B$ decays exponentially to approximately .5. For the remaining graphs, the final proportion of individuals with the true opinion is larger than the initial counter-campaign, showing the counter-campaign convinced non-committed individuals of their viewpoint. While these results suggest that the larger the counter-campaigns is, the better, our large counter-campaign was unable to keep the majority of individuals from holding the disinformation.

## Discussion

In this work, we investigated the spread of disinformation on various weighted, directed, heterogeneous graphs with committed minorities using the binary agreement model. We evaluated the effectiveness of three types of policies, namely content moderation, education, and counter-campaigns. For each policy we implemented two strategies to combat the spread of disinformation. These strategies were tested on hundreds of graphs based on 15 graph topologies, and a real social network generated from data of Asian users of LastFM^50^. Over 18,000 core hours were used to explore the effectiveness of the mitigation strategies on these networks.

Regarding content moderation, we found that removing nodes based on our influence metric did not significantly outperform removing nodes randomly for most graphs. As more nodes were removed, the two methods converged towards each other. This is encouraging because our simple influence metric scales as $O(|V||E| + |V^2|)$, which can quickly become burdensome to calculate for large graphs. However, on the real social network graph a more targeted approach outperformed a random one. Therefore, our model suggests that removing sources of disinformation as they are identified is a viable method for implementing content moderation, however a more targeted approach might prevail on graphs with unique topology.

We also explored education-based policies and found that a strategy that increased people’s skepticism had a notably stronger positive impact in our model than a strategy that focused on people’s attention to disinformation. In fact, our model suggested that strategies such as fact-checking, had little effect, and only showed a small positive effect when any change occurred. However, it is worth noting that our definitions for skepticism and attention are crude due to the simplicity of our model. Therefore, while our model suggests that strategies that bring attention to disinformation may not be very effective, we caution against blindly following this advice. We recommend implementing any method that can increase skepticism, while noting that strategies that focus on immediately biasing individuals towards the truth may not be as effective as those aimed at more broadly educating people to be critical thinkers.

After exploring the effectiveness of counter-campaigns, we found that small counter-campaigns were ineffective at combating the spread of disinformation for most graphs. When employed, they left only the original counter-group holding the true opinion. Contrarily, larger counter-campaigns were able to sway some uncommitted individuals, suggesting those committed to the disinformation had less reach. However, in the real social network, grid, and barbell graphs both large and small counter-campaigns resulted in an approximately equal split between individuals holding the true or disinformed opinion. We believe this is due to the topology of these graphs and our initialization of the committed agents. We initialized counter-campaigns and the original committed minority on opposite sides of graphs to account for them starting locally on the graph. However, in the real social network, grid, and barbell graph these two sides are more strongly intraconnected than interconnected, resembling echo-chambers. Thus each competing group of committed agents overtake their respective side of the graph, resulting in a fairly equal split of opinions. In contrast, previous work has explored initializing the competing committed group randomly on graphs^40^.

For each strategy we implemented based on content moderation, education, and counter-campaigns, we found that the fraction of individuals with the true opinion versus the fraction of individuals committed to the disinformation were quantitatively similar. The vast majority of individuals held the true opinion until a critical number of individuals were committed to the disinformation. Then there was a tipping point in opinions where the disinformation group quickly dominated the popular opinion. Past this critical value the number of people believing each opinion stays constant. The different implementations of our polices were able to quantitatively vary these features. Most notably, education and content moderation increased the required size of the minority needed to sway the majority and counter-campaigns preventing individuals from being swayed by disinformation. The different implementations of our polices were able to quantitatively vary these features through various mechanisms. The quantitative changes produced by these strategies are summarized in Table 3. Content moderation and education address two sides of the disinformation issue: the source and the receiver. Content moderation attempts to curtail the spread at its origin, while education, especially fostering skepticism, equips individuals to resist misleading narratives. On the other hand, counter-campaigns introduce a competitive narrative to challenge and diminish the influence of the primary disinformation source. Each strategy has its merits, but their effectiveness can vary based on the structure and nature of the network in question. Most notably, education and content moderation had stronger effects on the tipping point than content moderation. Specifically, skepticism moved the tipping point forward, while larger counter campaigns increased the number of individuals that remained with the true opinion after the tipping point. As the ability of generative models to produce convincing material grows they might be used to generate disinformation that is difficult to detect^10^. In such scenarios, educating individuals will become evermore important.

Based on the results of our simulations, education-based policies that increase skepticism and counter-campaign policies were the most promising. Examples of real world policies that increase skepticism could include creating media literacy programs. Such program teach individuals to recognize trustworthy sources and discern fact from opinion. Additionally, incorporating critical thinking curriculum into the education curriculum at an early age can build a foundation of skepticism. Another potential strategy would be creating feedback loops on social media platform that notify users when they engage with potential disinformation. Example of real world strategies that focus on counter-campaigns could range from launching corrective advertising campaigns that directly address and correct false narratives, to establishing rapid response teams. These teams would monitor social media for emerging disinformation so that they could quickly launch counter-efforts.

In summary, our goal in this work was to create a model that captures realistic mitigation strategies within the confines of a binary agreement model so that our result may be applicable to both modelers and policymakers. Additionally, there is a vast number of other policies, strategy implementations, and combinations of the two that could be explored. This leaves many avenues for future studies to extend our work. One promising direction is to incorporate a more realistic model of the spread of opinions, such as the attraction-repulsion model^36^. This model allows for multiple, continuous-valued opinions to be held by each individual. This more nuanced representation of opinions allow individuals to become more or less closely aligned with their neighbors. Additionally, we could enhance the realism of the model by incorporating multiple online and offline social networks, dynamic networks, and news sources. Beyond improving the model, future studies can explore policies we did not test, optimal parameters for existing policies, or learning entirely new policies.

### Supplementary Information

Supplementary Information.

## Data Availability

The datasets used and/or analysed during the current study are available from the corresponding author on reasonable request.
