# Supplementary material for: Mathematical modeling of disinformation and effectiveness of mitigation policies
Source: Sci Rep. 2023 Oct 31;13:18735. doi: 10.1038/s41598-023-45710-2 (PMC10618487; doi:10.1038/s41598-023-45710-2)
Supplement: Supplementary file 1 — Supplementary Information. [file 41598_2023_45710_MOESM1_ESM.pdf]

Supplement to Mathematical Modeling of Disinformation and Effectiveness of Mitigation Policies

1 DeGroot Model

In this Supplement, we analyze and adapt a second model, the DeGroot model,[1] to each of the policies discussed in the main document. The DeGroot model was chosen for its mathematical transparency, with the goal of assessing its ability to describe the effects of disinformation-mitigation policies. In Subsection 1.1, we review the basic properties of the DeGroot model. In the following subsections, the model is then modified to include disinformation-mitigation strategies. Section 2, Section 3, and Section 4 contain figures illustrating the effects of content moderation, education, and counter-campaigns, respectively, in this model.

1.1 DeGroot Model

The model employed in the main document requires moderately intensive computational effort to explore the wide ranges of anti-disinformation policies, their model implementations and social graphs. It would be convenient to have an analytic model of the spread of disinformation that provides insight and intuition with minimal computational effort. A model that employs a linear update, contains social-network structure and has an equivalent of a committed agent is the DeGroot model.[1] We adapt this model to include the three interventions policies (education, content moderation, and counter campaigns) we consider in this work and their effects on disinformation dynamics.

In the DeGroot model, we consider a group of N agents connected by a network, which can be represented by an adjacency matrix $\mathbf{A} = [a_{ij}]$. Each agent forms its belief as a weighted average of the beliefs of its neighbors. Formally, if $\mathbf{x}(t) = [x_1(t), x_2(t), \ldots, x_N(t)]^T$ represents the beliefs of the N agents at time t, then the belief of agent i at time $t+1$ is given by the discrete-time update

$$x_i(t+1) = \sum_{j=1}^{N} t_{ij} x_j(t), \tag{1}$$

or in matrix notation,

$$\mathbf{x}(t+k) = \mathbf{T}^k \mathbf{x}(t). \tag{2}$$

Here, $\mathbf{T} = [t_{ij}]$ is referred to as the trust matrix, with t_{ij} representing the (constant) weight that agent i assigns to agent j when updating its belief; in other words, t_{ij} quantifies the extent to which agent i trusts agent j. The trust matrix $\mathbf{T}$ is derived from the adjacency matrix A by normalizing each row to sum to one; i.e., $t_{ij} = a_{ij} / \sum_{k=1}^{N} a_{ik}$. Starting from some initial set of beliefs $\mathbf{x}(0)$, this model repeatedly updates each agent's belief based on a weighted sum of all other agents' beliefs, weighted by levels of trust.

In this framework, we have an interpretable update rule in which t_{ij} represents a trust level that varies from $t_{ij} = 0$ for no trust to $t_{ij} = 1$ for complete trust; the model can be initialized to these discrete values, or trust levels can be allowed to be continuous. In Fig. 1, we show examples of symmetric, directed graphs with equal weights, and their corresponding trust matrices. In general, however, the graph represented by $\mathbf{A}$ is weighted and directed: I may listen to you, but you need not listen to me. It is through flexibility in $\mathbf{T}$ that we include committed agents and mitigation strategies in the DeGroot model; in the context of the DeGroot model, committed agents are referred to as "stubborns".[2]

Many details of the model dynamics are easily revealed by considering the structure of $\mathbf{T}$. By construction, $\mathbf{T}$ is a right-stochastic matrix and thus has a spectral radius of 1; this is an important property, as updates are obtained using progressively higher powers of $\mathbf{T}$, as in (2). At least one eigenvalue is 1 and corresponds to a right eigenvector whose elements are all 1. Eigenvalues associated with left and right eigenvectors are equal for square matrices; this implies that T has at least one left eigenvector associated with an eigenvalue of 1. If $\mathbf{T}$ can be represented as a directed, weighted graph that is strongly connected (i.e., irreducible), then we can apply the Perron-Frobenius theorem that states that there is a unique left eigenvector $\mathbf{v}$ that sums to one and corresponds to an eigenvalue of 1. This eigenvector determines the final consensus belief; specifically, the final state is given by

$$x_{\text{final}} = \mathbf{v}^T \mathbf{x}(0), \tag{3}$$

where we see that $\mathbf{v}$ represents the long-term influence of the agents. This equation identifies $\mathbf{v}$, which is the eigenvector centrality of $\mathbf{A}$, as the relevant parameter for describing the spread of information, including disinformation. Intuitively, eigenvector centrality measures a node's influence in a network by considering both the number and quality of its connections,

recursively factoring in the influence of neighboring nodes. It is also immediately clear from (3) that initial beliefs $\mathbf{x}(0)$ contribute to the final consensus. A block structure in $\mathbf{T}$ represents independent echo chambers that evolve according to the reducibility class of each block. It is possible that $\mathbf{T}$ is periodic, although some structures (e.g., upper/lower triangular or symmetric) guarantee that it is aperiodic. We can also examine the second largest eigenvalue of $\mathbf{T}$ to obtain the eigenvalue gap, which reveals the speed of convergence; larger gaps are associated with faster convergence.

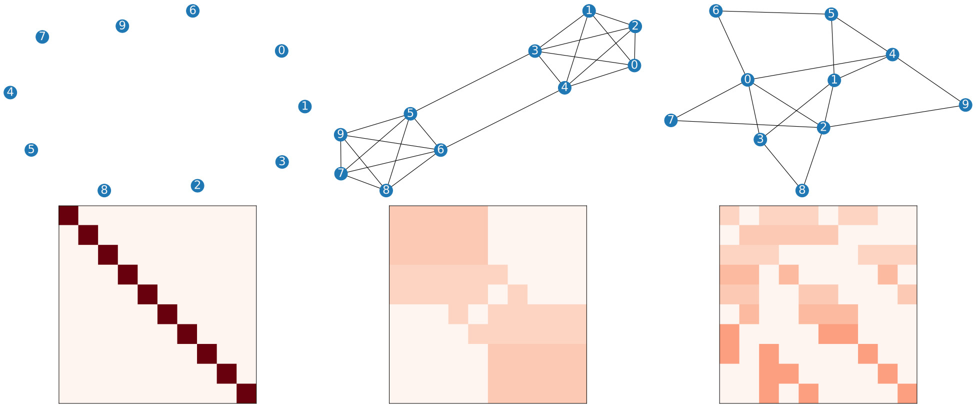

Figure 1. Example graphs and their corresponding trust matrices. Despite its simplicity, the DeGroot model contains a complete social network structure as a directed and weighted graph. Social network graphs are shown in the top row, and their corresponding trust matrices are shown in the bottom row.

We now extend the DeGroot model to include the three disinformation-mitigation strategies – education, content moderation, and counter campaigns – that we consider in this work and their effects on the spread of disinformation in the presence of stubborns. We model these interventions as modifications to the trust matrix and/or the initial condition and analyze their impact through the lens of the eigenvalue spectrum and eigenvectors of the modified trust matrix. For each mitigation strategy, we begin with a (scale-free) Barabasi-Albert graph with 100 nodes. Each edge is then converted into two directed arcs that point in opposite directions, and their weights are determined using social transitivity (described in the main document). This method of generating directed and weighted edges guarantees that the corresponding trust matrix is irreducible, because the graph is clearly strongly connected. Opinions have values between 0 and 1; here, we assume that 0 is the truth and that 1 is disinformation. Values between 0 and 1 indicate a bias towards the truth or disinformation, with the exception of 0.5, which indicates a completely neutral position.

Stubborns are added to the model by selecting an individual i and setting $t_{ii} = 1$, with $t_{ij} = 0$ for $i \neq j$. Importantly, the addition of stubborns make the trust matrix reducible, and consensus is not necessarily reached. However, opinions will converge to a value that depends on the structure of the trust matrix and on agents' initial opinions. One important scenario is that in which all stubborns have the same opinion. In such a case, any node that is reachable from a stubborn will converge to the stubborn's opinion. Using our method for generating trust matrices guarantees that the presence of one or more stubborns with the same opinion, and no stubborns with a different opinion, will eventually converge the network to a consensus of their opinion. Unless otherwise stated, $x_i(0) = 1$ if an individual is stubborn, and is initially 0 otherwise, in each of our scenarios; i.e., stubborns are committed to disinformation, and all other agents initially believe the truth. The goal of mitigation strategies is to minimize x_{final} when a consensus is reached, or if a consensus is not reached, then the goal is to push opinions towards the truth or to minimize the eigenvalue gap when opinions tend towards disinformation.

1.2 Education

Skeptical and attentive education strategies are modeled by altering the t_{ij} based on the type of education received by each agent i. In the skeptical education strategy, the values of the diagonal elements of $\mathbf{T}$ of committed agents are decreased; this reflects a weaker commitment to one's own belief. In the attentive strategy, the t_{ij} of neighbors adjacent to stubborn agents are decreased. To test both education strategies, utilized Barabási-Albert graphs with 100 agents, selecting 10 of these agents to be committed to opinion A, leaving the remainder uncommitted to opinion B. The edges of these graphs are then transformed into two directed edges in both directions, weighted using social transitivity as described in the main document. We generate a trust matrices, $\{\mathbf{T}\}$ by normalizing the adjacency matrices of these graphs, and then we apply our education mitigation strategies to these trust matrices.

The skepticism educational strategy reduces diagonal elements t_{ii} of the trust matrix by a factor $\sigma < 1$, and the remaining elements corresponding to a committed individual's neighbors (determined by the adjacency matrix) are increased by equal

parts of σt_{ii}, so that the row still sums to 1. Such a change to $\mathbf{T}$ changes the eigenvalue spectrum. Most importantly, it guarantees that $t_{ii} < 1$, ensuring that the matrix is reducible, and a consensus opinion is reached at a value less than 1. In Fig. 2, we show the consensus opinion versus σ. For each value of σ, we executed 50 simulations and averaged over initial conditions and graphs; for each value of σ, the average consensus opinion reached is shown with a black $\mathbf{x}$, and the value each simulation converged to is shown with a grey dot. When there is no education ($\sigma = 0$), the stubborns' opinion takes over, and all simulations converge to disinformation. As the stubborns become more skeptical (σ increases), the average value of consensus decreases and approaches a constant value of approximately 0.2. The variance in the consensus value is approximately constant for all values of $\sigma > 0$.

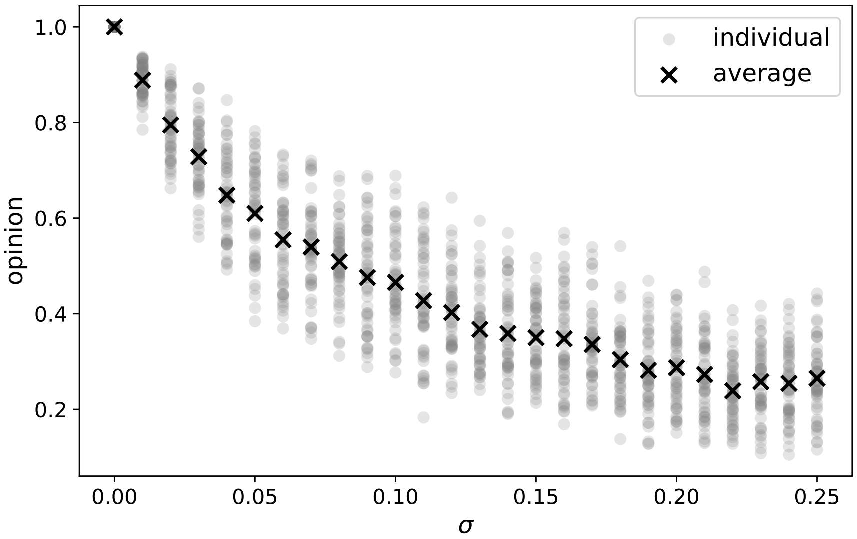

Figure 2. Final consensus opinions versus the education parameter σ. For each value of σ, gray circles and dark $\mathbf{x}$ indicate the value each individual run converged to and the average final opinion over 50 runs, respectively. The final opinion decreases toward the truth with more skepticism education (larger values of σ).

The attentive strategy is modeled by increasing elements t_{ij} that correspond to non-stubborn individuals i listening to other non-stubborn individuals j with initial opinions of zero, by a factor $\alpha > 1$, and reducing elements $t_{i,k}$ equally such that the rows of $\mathbf{T}$ sum to 1. Such a change biases individuals to listen more to true opinions. The attentive educational strategy does not affect the opinions of stubborn individuals, which means that eventually, the population will still converge to the disinformation opinion. However, the attentive strategy can affect how quickly the model converges, which is measured by the eigenvalue gap. A smaller eigenvalue gap is associated with a longer convergence time. In Fig. 3, we examine the eigenvalue gap versus α in 50 simulations. The blue line shows the average eigenvalue gap in the absence of a mitigation strategy, and the orange line shows the average eigenvalue gap when long-term education is employed. The 95% confidence intervals for both are shown as shaded regions. The attentive education mitigation strategy was applied to the same 50 networks examined in the absence of a mitigation strategy, and thus, both lines share random fluctuations. When no mitigation is applied, the eigenvalue gap is fairly constant with respect to α. Attentive education reduced the eigenvalue gap slightly as α increases, but not substantially.

The results for the skepticism and attentive education strategies we obtained using a DeGroot model are consistent with what we found when studying such strategies using the binary agreement model. As we found using that model, reducing people's commitment to disinformation can largely counteract the stubborn's ability to sway individuals' opinions. However, solely biasing people towards the truth is not sufficient to overcome the influence of stubborns.

1.3 Content Moderation

In our model of disinformation spread, we first create a Barabási-Albert graph with 100 agents, selecting 10 of these agents to be committed to opinion A, leaving the remainder uncommitted to opinion B. The edges of this graph are then transformed into two directed edges in both directions, weighted using social transitivity as described in the main document. We create three copies of this graph for distinct analyses. In the first copy, we randomly select n committed nodes to remove, where n

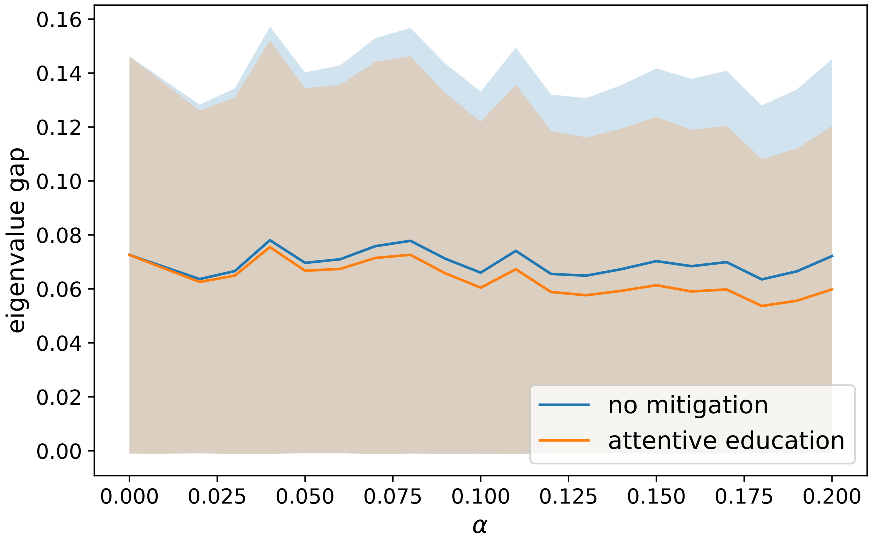

Figure 3. Eigenvalue gap versus the education parameter α. The blue line shows the eigenvalue gap when no education is employed, while the orange line shows the eigenvalue gap for increasing amounts of attentive education. The eigenvalue gap reduces slightly as α increases meaning the rate that the model converges to the disinformation is slightly slowed.

varies from 0 to 9. In the second copy, we systematically remove n committed agents based on their eigenvector centrality. To do this, we compute eigenvector centrality, remove the node with the highest centrality, recompute the centrality, and repeat this process until n agents have been removed. The third copy remains unaltered, serving as a control. For each of these three graphs, we generate a trust matrix by normalizing the adjacency matrix and then calculate the eigenvalues of the trust matrices. We also compute the difference in the two largest eigenvalues, ignoring any repeated values (e.g., if the eigenvalues are 1, 1, 0.75, then the eigenvalue gap is 0.25). This entire process is repeated 50 times. For each value of n, ranging from 0 to 9, we average the 50 runs and plot this average as a solid line, also calculating a 95% confidence interval to create a band around the solid lines. This methodology allows us to explore the effects of content moderation through both random and strategic removal of influential nodes on the dynamic behavior of the system, as characterized by the eigenvalue gap. Removing individuals from the graph alters the dominant eigenvector and the eigenvalue spectrum of $\mathbf{T}$, thereby shifting control away from the removed agents. However, as discussed previously, if we do not remove all of the stubborns, then the population opinion will eventually converge to the disinformation opinion. As before, we can then examine the eigenvalue gap to determine whether the rate of convergence changes.

In Fig. 4, we show the eigenvalue gap versus the number of committed individuals removed from the model. Results for removing individuals randomly are shown with a solid orange line, and results for removing individuals based on their level of influence are shown with a solid green line. We also show results for removing no individuals with a solid blue line, for comparison. The results shown with all three solid lines were obtained using the same graphs and are averages over 50 simulations. The shaded regions indicate 95% confidence intervals. When no individuals are removed, the eigenvalue gap is approximately 0.07. When stubborns are removed from the graph with either approach, the eigenvalue gap tends towards zero. However, when stubborns are removed based on eigenvalue centrality, the eigenvalue gap converges towards zero faster than when stubborns are removed randomly. Therefore, in this model, a targeted approach to removing stubborns slows the spread of disinformation more than does removing individuals randomly.

1.4 Counter Campaigns

Counter campaigns are modeled by introducing another group of stubborns who are committed to the truth. The social networks we consider contain 3 subgroups of agents: 1) agents spreading disinformation (n_A nodes), 2) agents running a counter campaign for truth (n_B nodes), and 3) regular agents comprising the remaining nodes. We model the committed agents as "stubborn" in the DeGroot model by setting their self-weights to 1. This ensures that they do not update their opinions over time. The initial opinion vectors for the groups are $x_i(0) = 1$ if you are committed to A, and 0 otherwise.

In Fig. 5 we show many examples of counter-campaigns of various sizes. As in our previous simulations, we began with

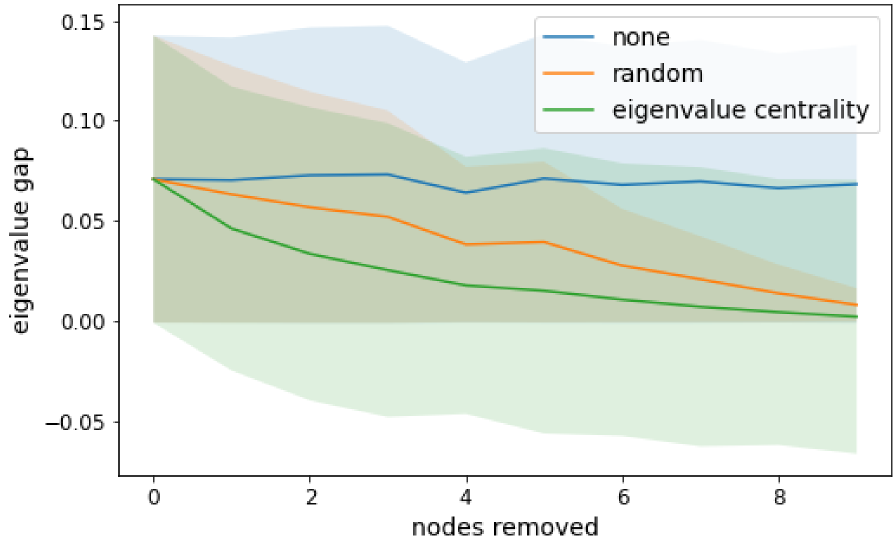

Figure 4. Eigenvalue gap versus number of committed agents removed. The eigenvalue gap versus removing agents randomly is shown in orange, and for removing agents based on eigenvalue centrality in green. As a baseline we show the eigenvalue gap when not removing agents in blue. Solid lines are the average of 50 simulations, while shaded regions are 95% confidence intervals. Removing agents with either strategy reduces the eigenvalue gap, however, a targeted approach based on eigenvalue centrality reduces the eigenvalue gap a a faster rate.

a Barabási-Albert graph with 100 agents, selecting 10 of these agents to be committed to opinion A, and n_B agents to be committed to B, and the remaining to be uncommitted to B. The edges of this graph are then transformed into two directed edges in both directions, weighted using social transitivity. Each subplot of Fig. 5 shows results for a values of n_B between 0 and 10, i.e., for a different size of counter-campaign. In each subplot, each individual's opinion versus the number of iterations is shown as a grey line. The committed agents' opinions can be seen as horizontal lines with values 0 and 1. Uncommitted individuals' opinions begin at zero and grow quickly to values between 0 and 1. As discussed previously, when all of the stubborns are committed to disinformation, all individuals eventually become committed to the disinformation opinion, as can be seen in the upper left subplot. However, as the size of the counter-campaign grows, uncommitted agents' opinions converge to opinions that are closer to the truth. When $n_B = n_A$, uncommitted agents' opinions converge to values near 0.5.

1.5 Conclusion

Understanding and combating the spread of disinformation in social networks is a critical challenge. Through the lens of the DeGroot model, we have examined three potential interventions and their impacts on the dynamics of belief formation. Our results highlight the importance of network topology and the distribution of trust in shaping these dynamics. In comparison with the more complete model in the main document, the DeGroot model allows for extremely fast exploration of parameter space, mainly related to the cost of diagonalizing T.

The results we obtained using the DeGroot model resemble the results of the binary agreement model in the main document. Namely, a skepticism education strategy was by far the most effective way to combat disinformation, while an attentive one had little to no affect. In our content moderation policies, we again saw if we do not remove all of the committed agents, eventually the committed agents will convince everyone of their opinion. However, in the DeGroot model our targeted approach performed better than our random approach, however, they both performed similarly. Counter-campaigns seemed to have a stronger effect in the DeGroot model compared to in the binary agreement model. However the initial positions of the counter-campaign agents were placed randomly when exploring the DeGroot model, which possibly gives those agents a further reach than those conisdered in the binary agreement model.

The DeGroot model allowed us to quickly explore some of our mitigation strategies, but its simplicity limits the applicability of results derived from using it. Utilizing the binary agreement model in the main document allowed us to incorporate more complex attributes, such as nonlinearities. Nonetheless, further research is needed to develop more sophisticated models and strategies for combating disinformation in social networks.

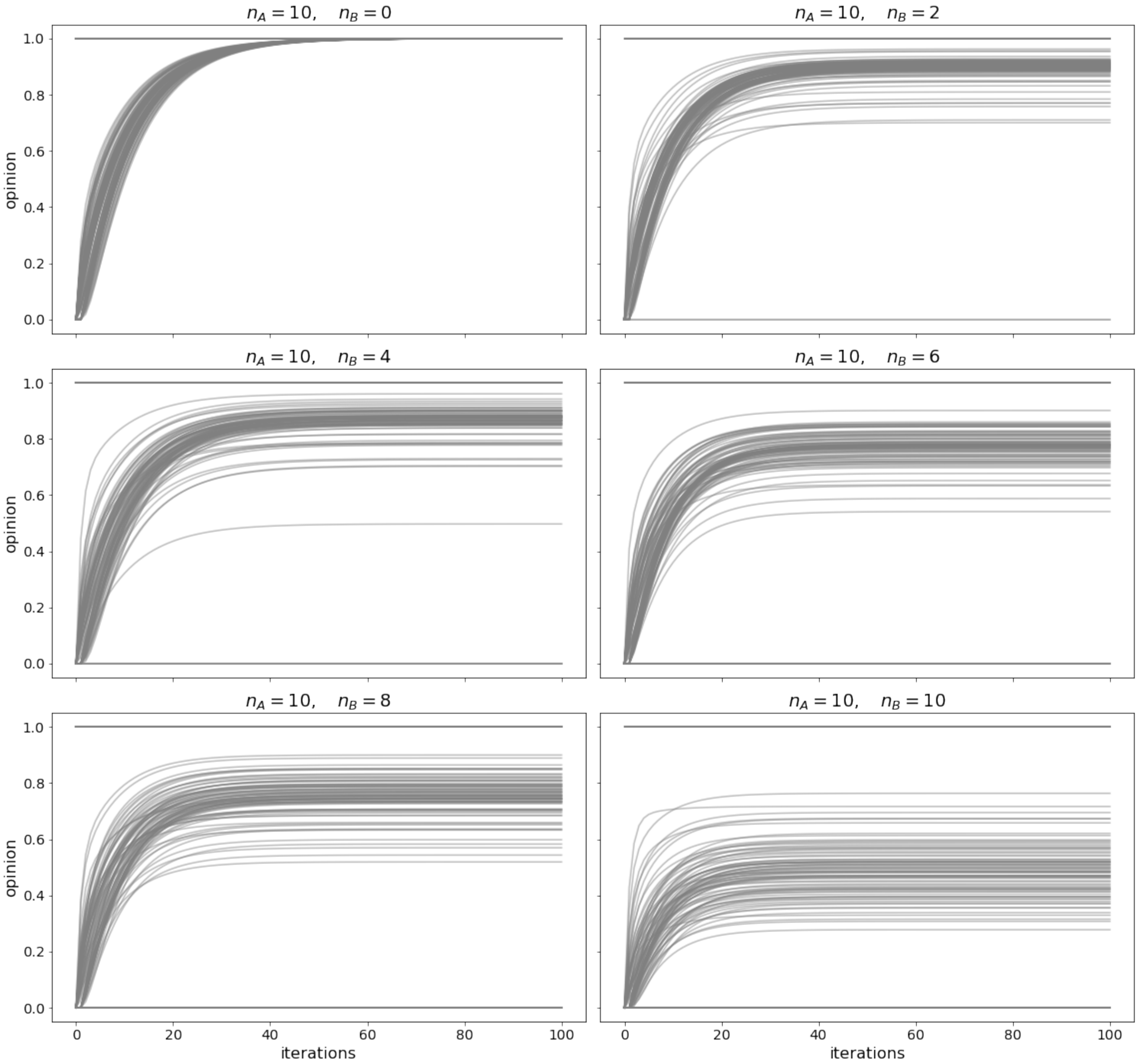

Figure 5. Agents opinions versus time in the presence of counter-campaigns. Agent's opinions versus the number of iterations of the DeGroot model are shown in grey for various sizes of counter-campaigns. For each size of counter-campaign (n_B), the size of the minority committed to the disinformation is held constant ($n_A = 10$). As the counter-campaign grows, uncommitted agents converge to opinions closer to the truth. When the counter-campaign is equal in size to the minority committed to disinformation, uncommitted agents converge to opinions near .5.

2 Content Moderation Figures

This section contains all of the results for content moderation applied to various artificial social networks. Each plot corresponds to the graph type listed in the caption. Results for removing nodes based on their level of influence are shown with solid lines, and those for removing nodes randomly are shown with dashed lines. The colors of the lines represent the percentage of the total nodes that were removed. Each opaque line shows the average of 5 simulations, and each transparent line shows results for a single simulation.

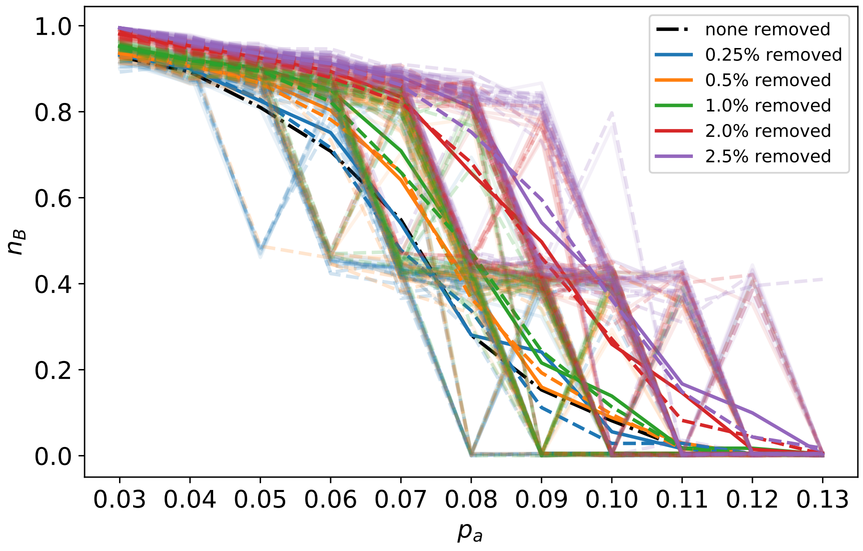

(a) Barbell graph

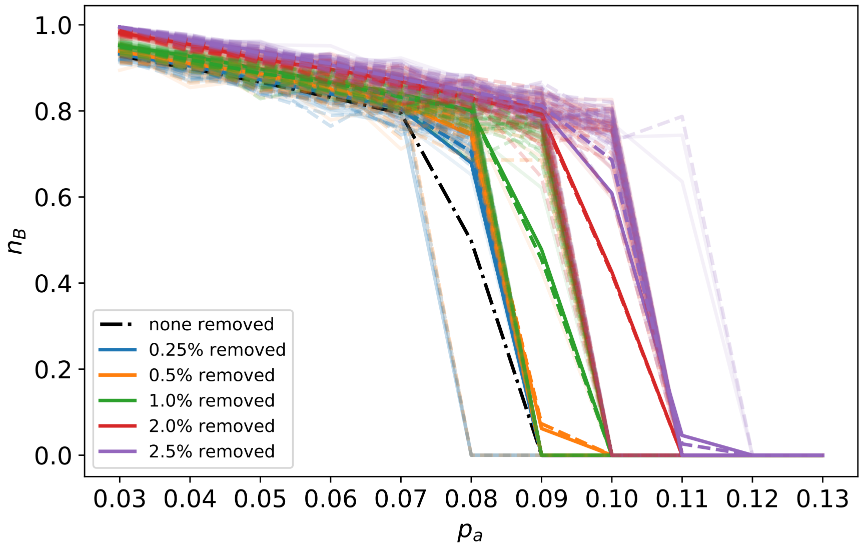

(b) Complete graph

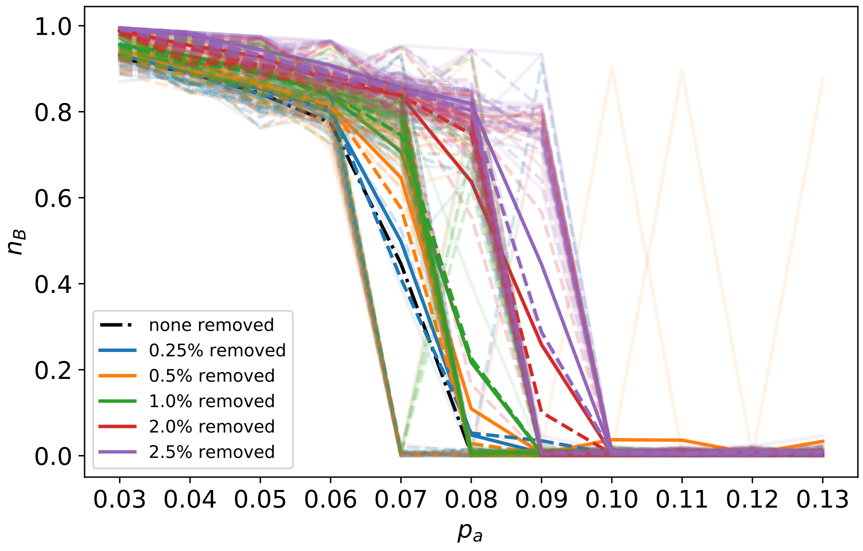

(c) Erdos-Renyi graph with $p = .02$

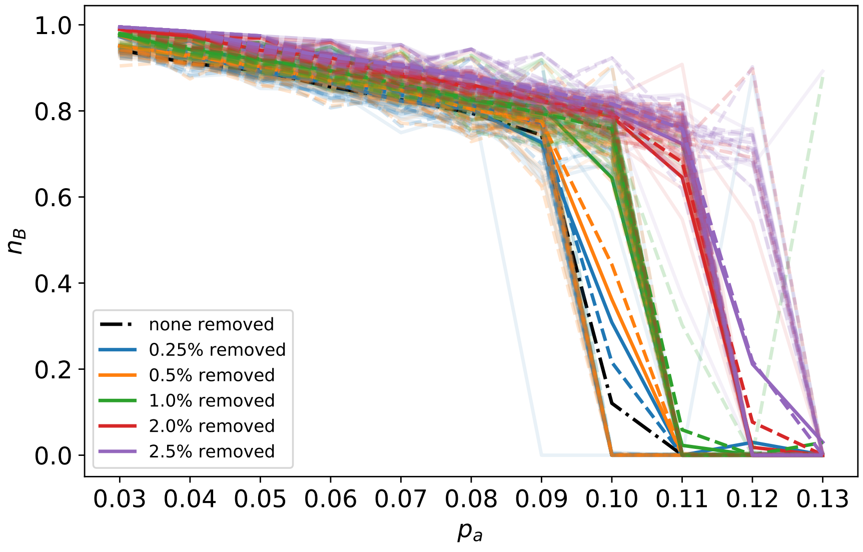

(d) Erdos-Renyi graph with $p = .12$

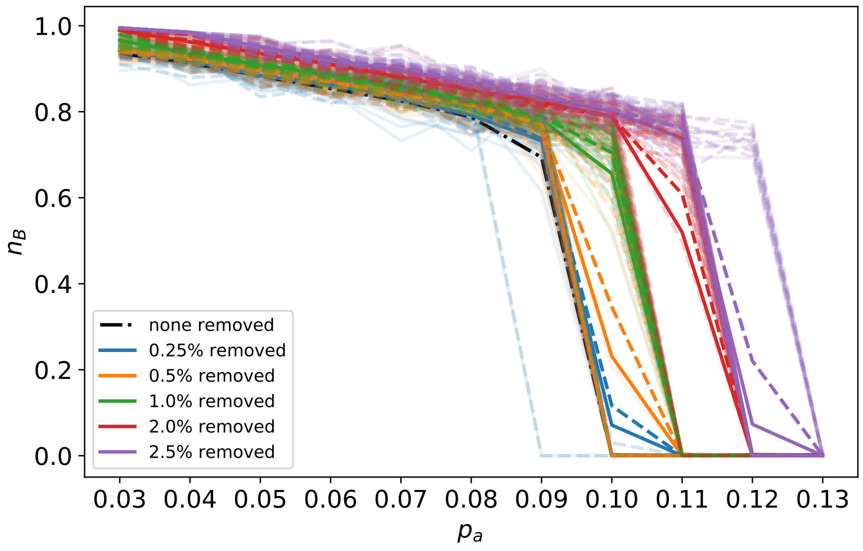

(e) Erdos-Renyi graph with $p = .25$

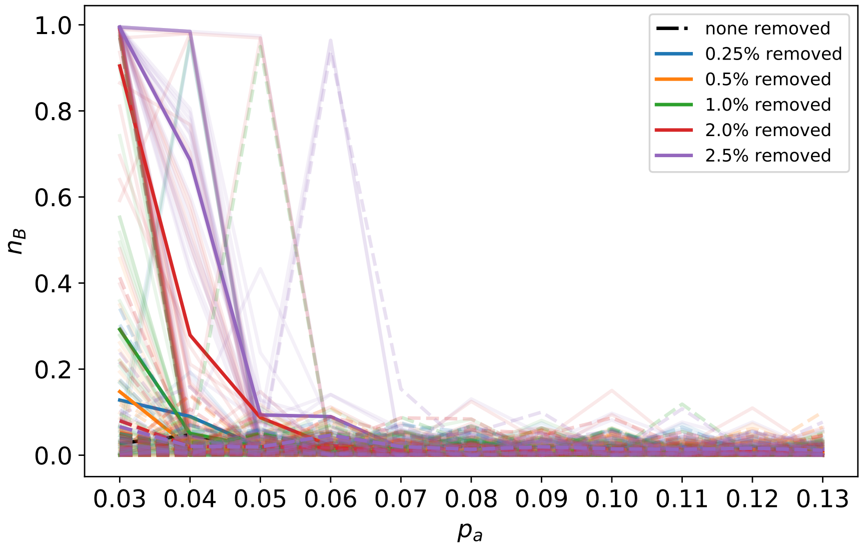

(f) Grid graph

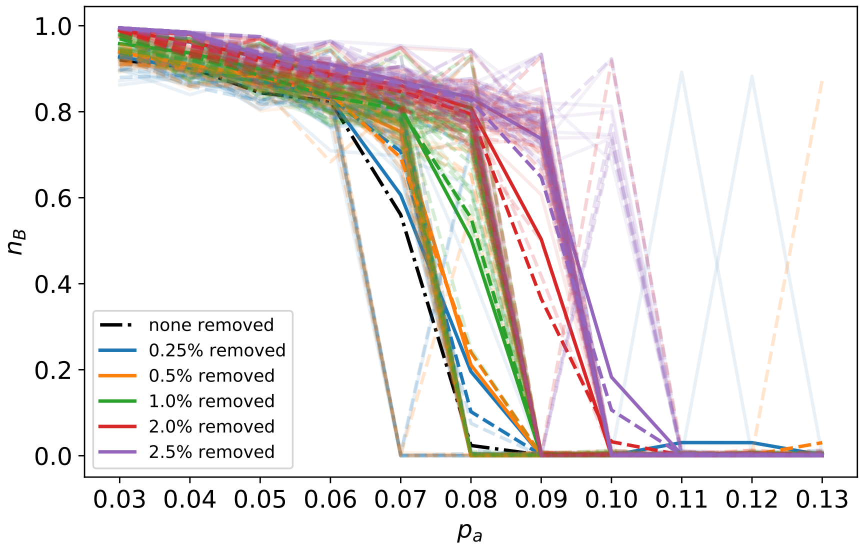

(g) Watts-Strogatz random graph with $k = 8$ and $p = 1$.

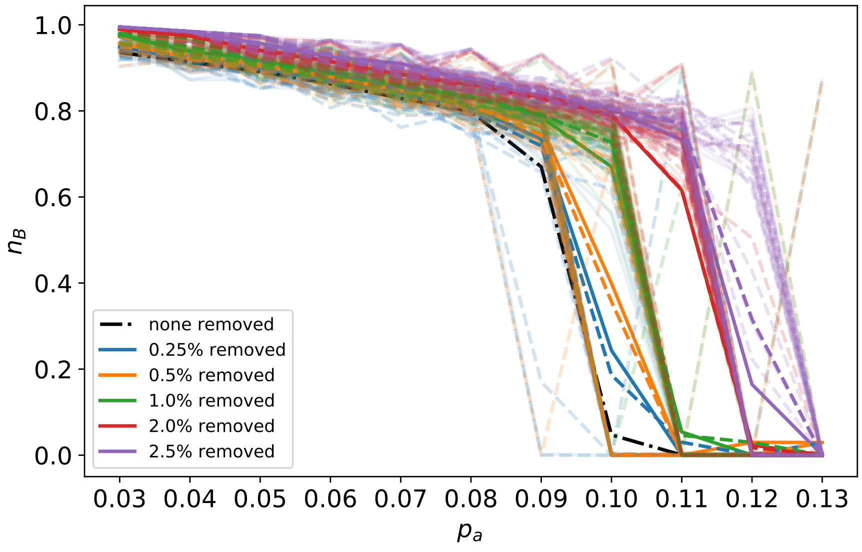

(h) Watts-Strogatz random graph with $k = 48$ and $p = 1$.

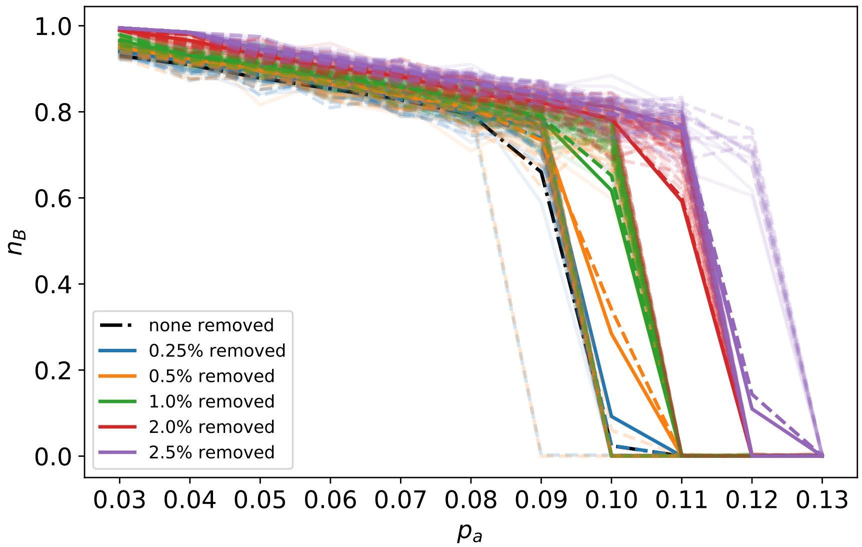

(i) Watts-Strogatz small world graph with $k = 100$ and $p = 1$.

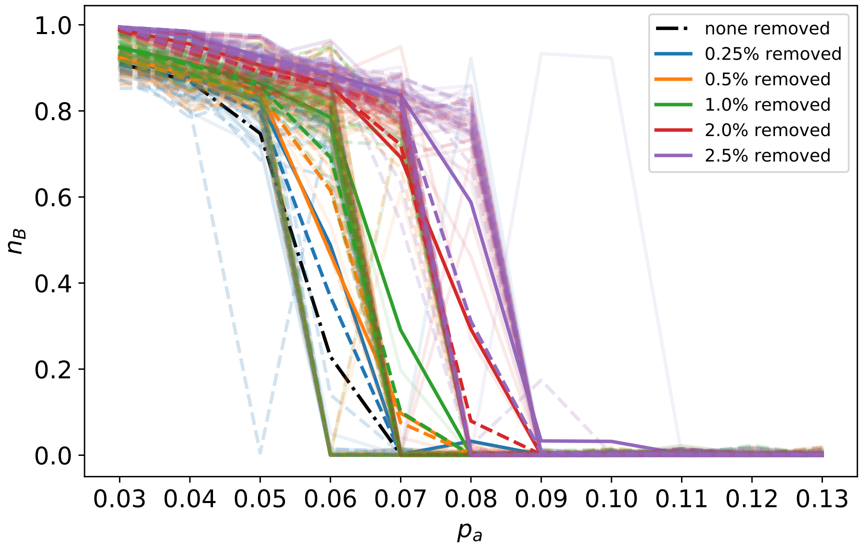

(j) Watts-Strogatz small world graph with $k = 8$ and $p = .5$.

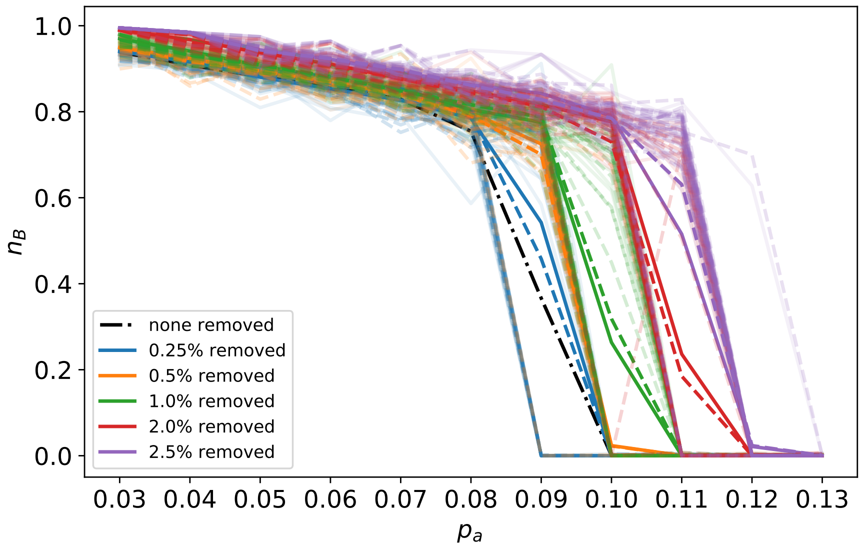

(k) Watts-Strogatz small world graph with $k = 48$ and $p = .5$.

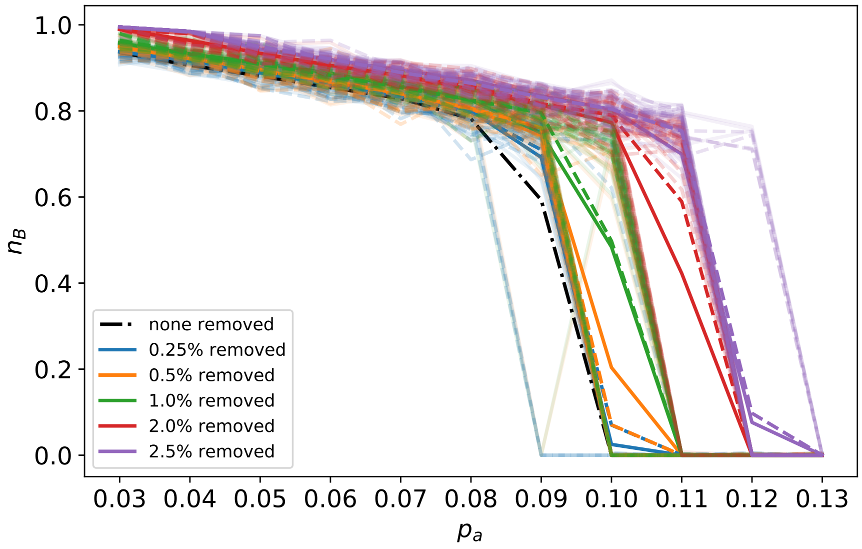

(l) Watts-Strogatz small world graph with $k = 100$ and $p = .5$.

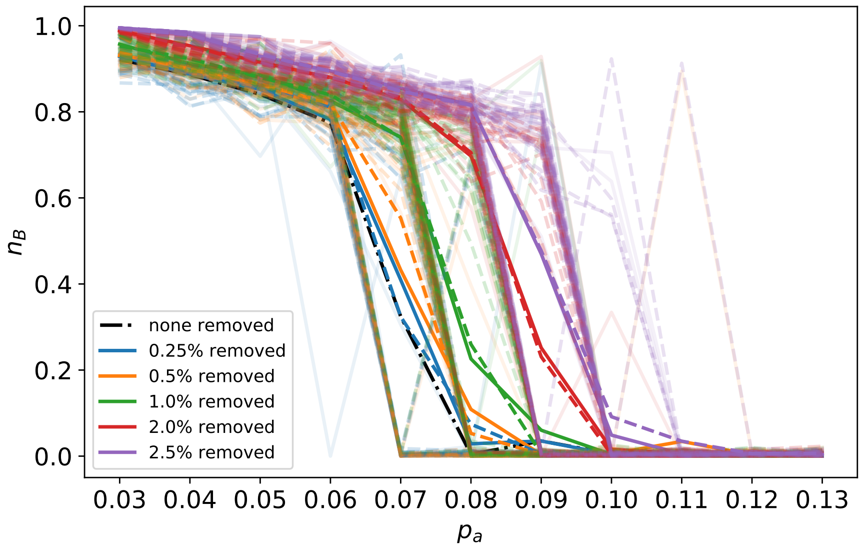

(m) Barabasi-Albert graph with $m = 4$.

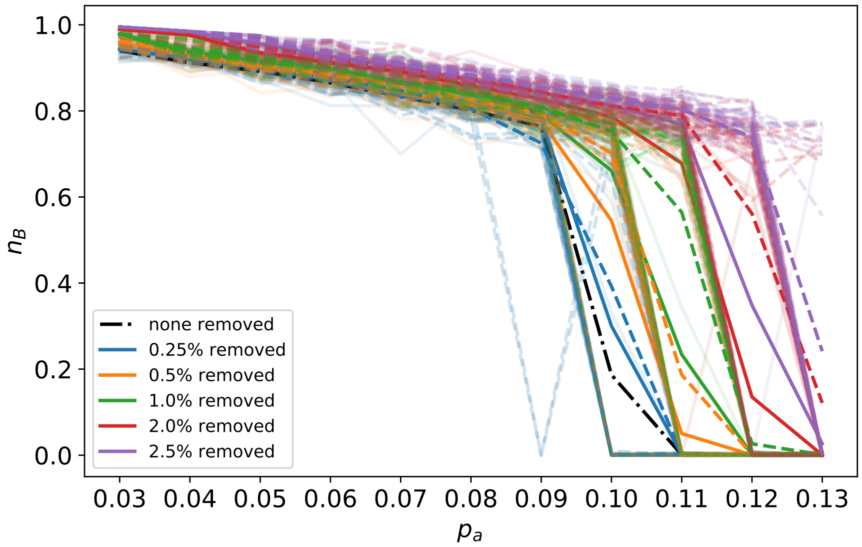

(n) Barabasi-Albert graph with $m = 24$.

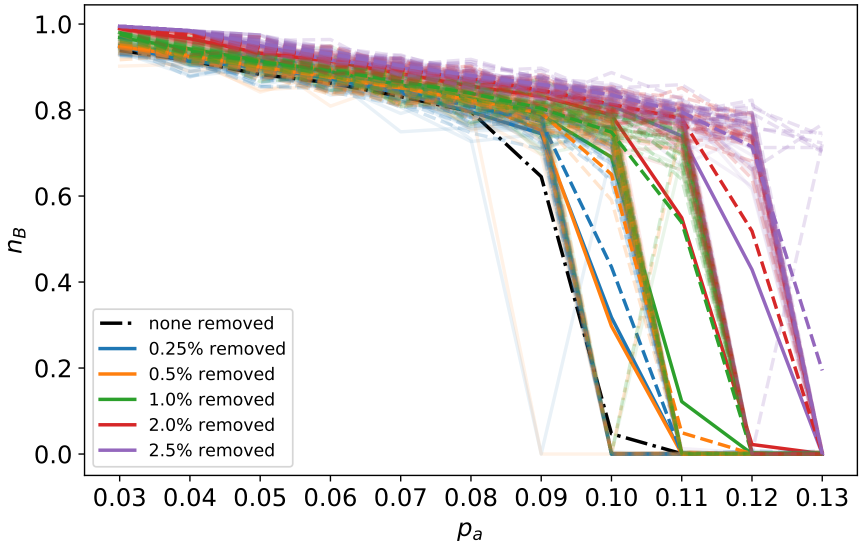

(o) Barabasi-Albert graph with $m = 50$.

3 Education Figures

This section contains all of the results for applying early and late education to various artificial social networks. Each plot corresponds to the graph type listed in the caption. We show the effects of early education with an orange dashed line, late education with a green dotted line, a combination of both with a solid blue line, and no education with a black dot-dashed line. Each opaque line shows the average of 30 runs, and transparent lines show results for individual runs.

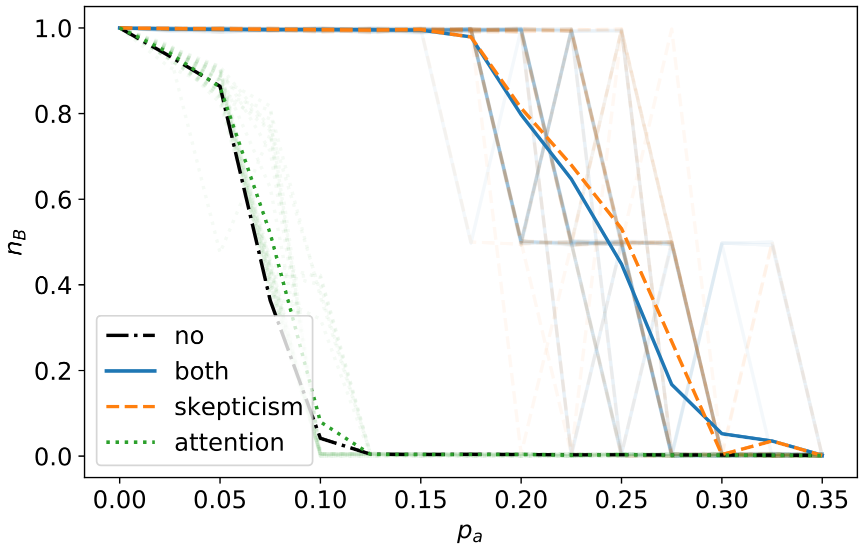

(a) Barbell graph

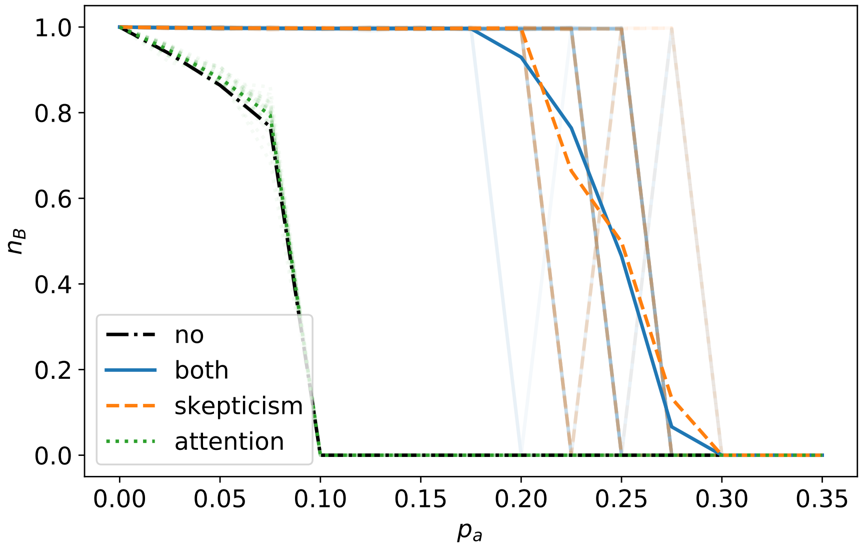

(b) Complete graph

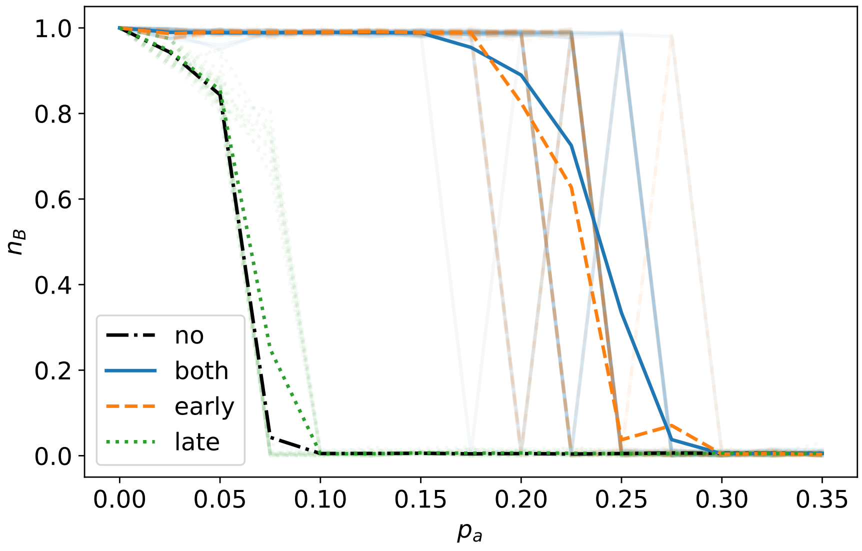

(c) Erdos-Renyi graph with $p = .02$

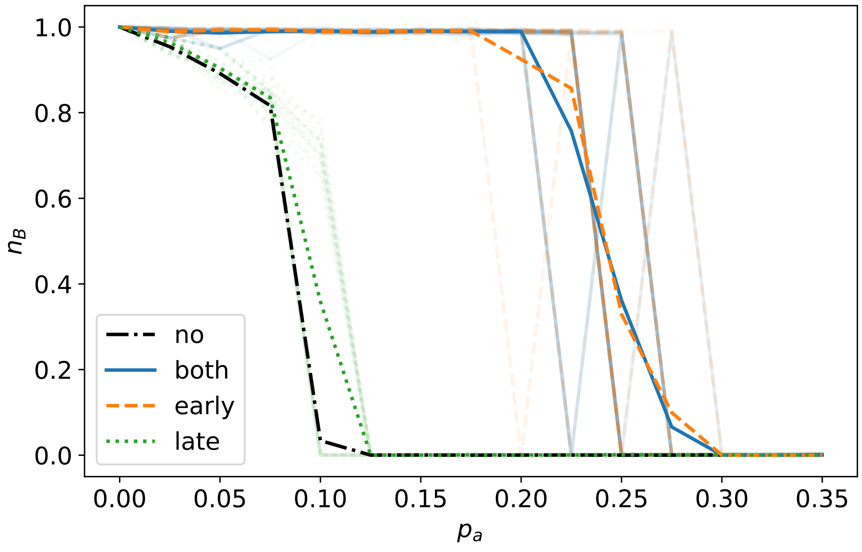

(d) Erdos-Renyi graph with $p = .12$

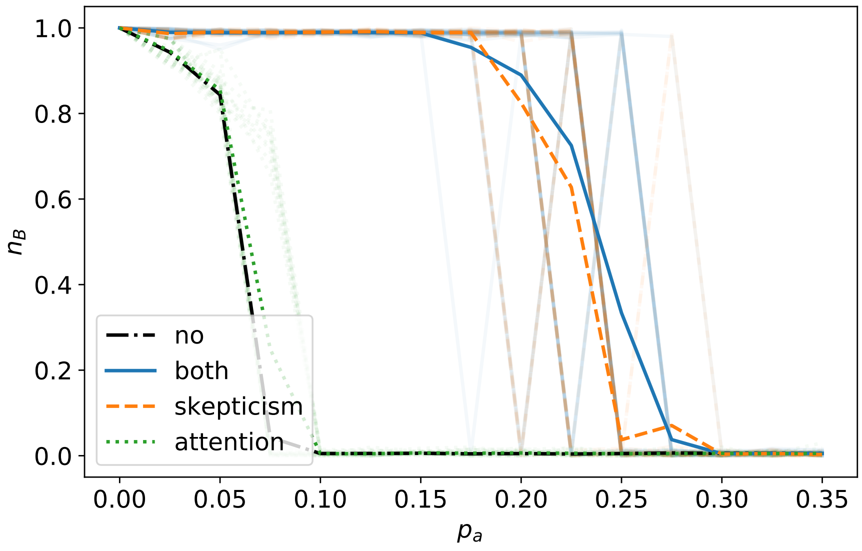

(e) Erdos-Renyi graph with $p = .25$

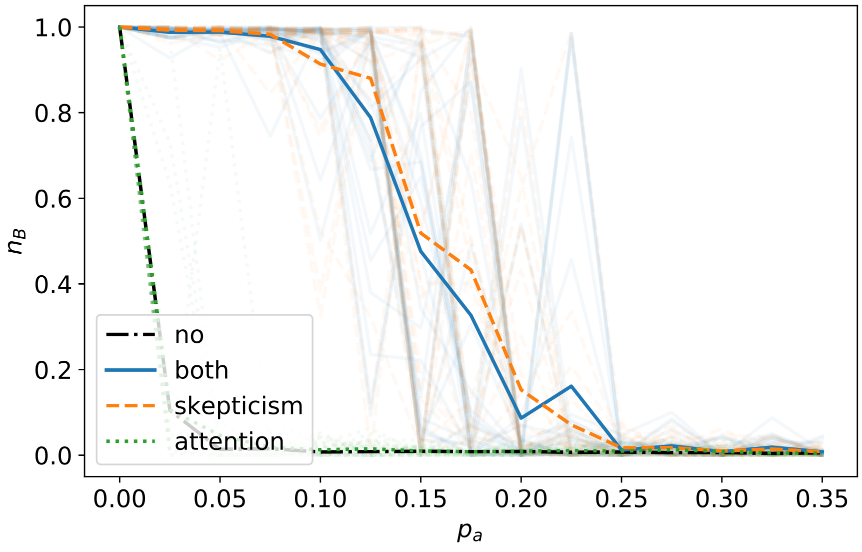

(f) Grid graph

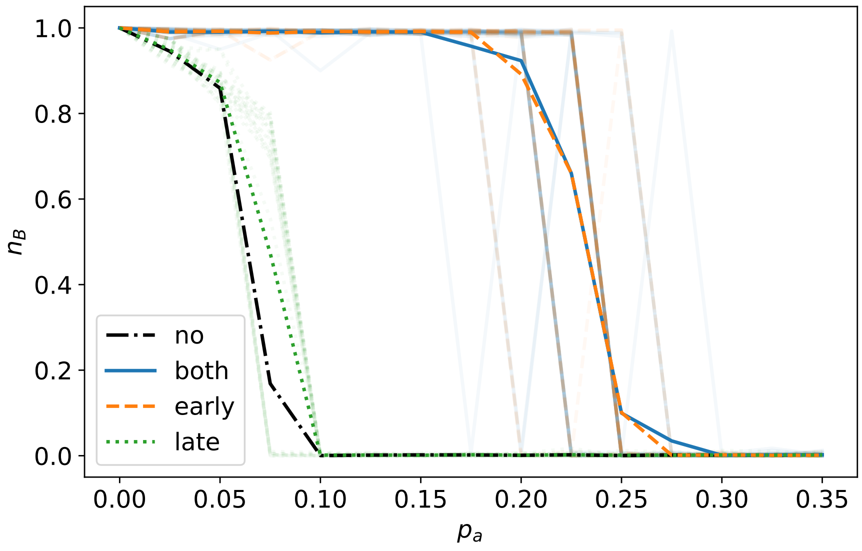

(g) Watts-Strogatz random graph with $k = 8$ and $p = 1$.

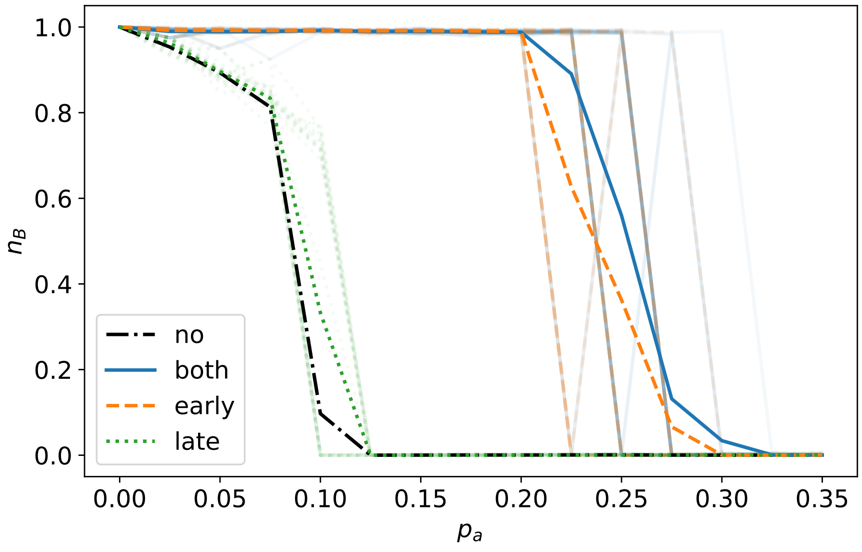

(h) Watts-Strogatz random graph with $k = 48$ and $p = 1$.

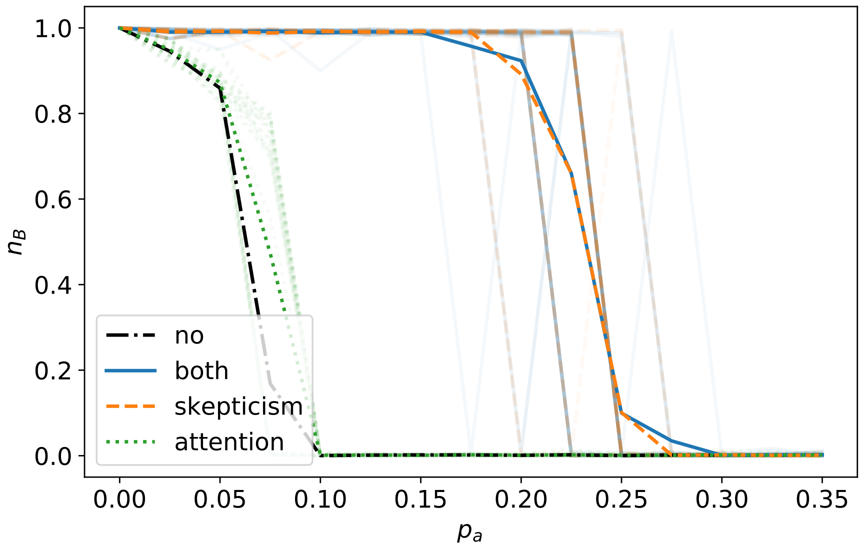

(i) WSR25

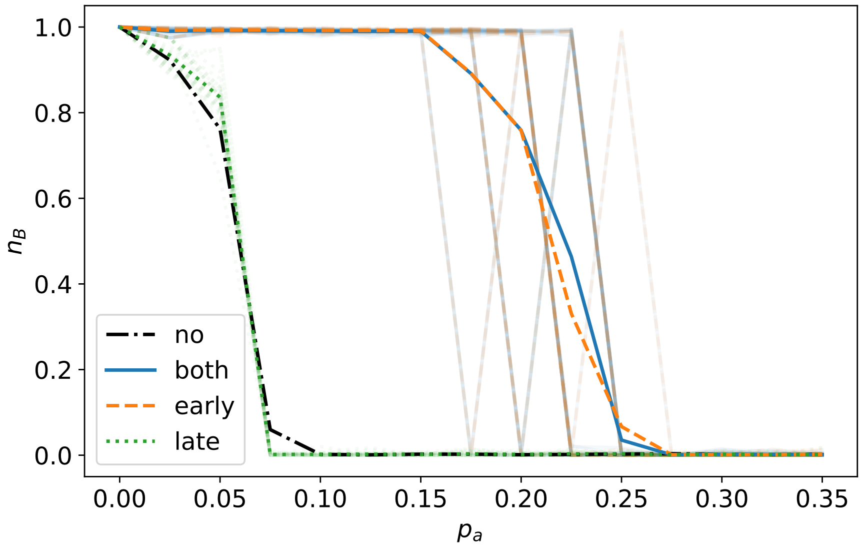

(j) Watts-Strogatz small world graph with $k = 8$ and $p = .5$.

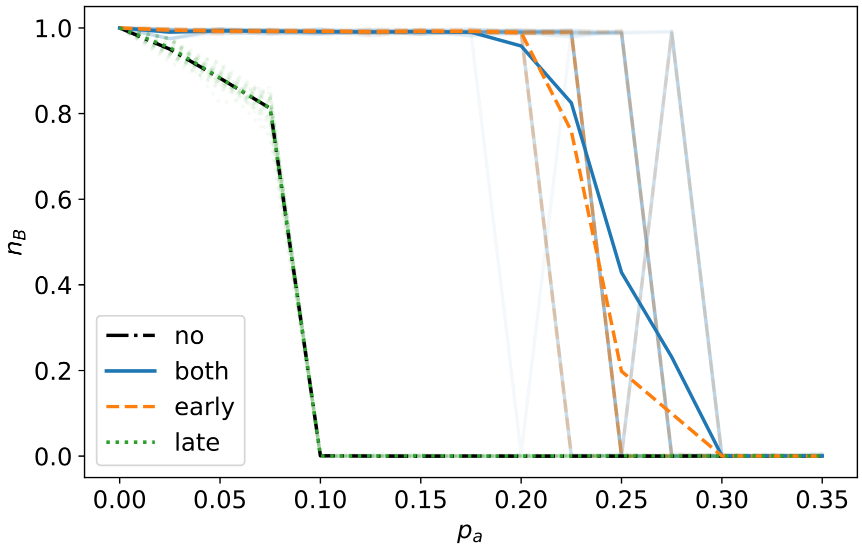

(k) Watts-Strogatz small world graph with $k = 48$ and $p = .5$.

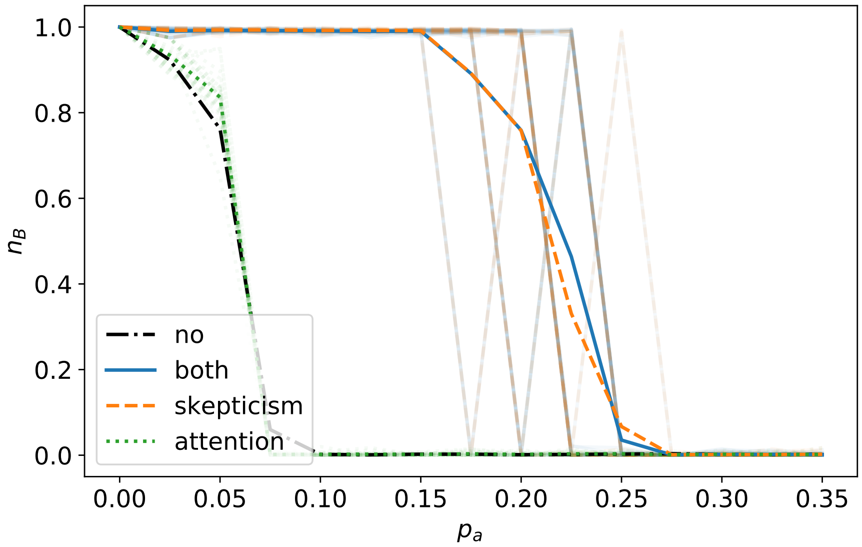

(l) Watts-Strogatz small world graph with $k = 100$ and $p = .5$.

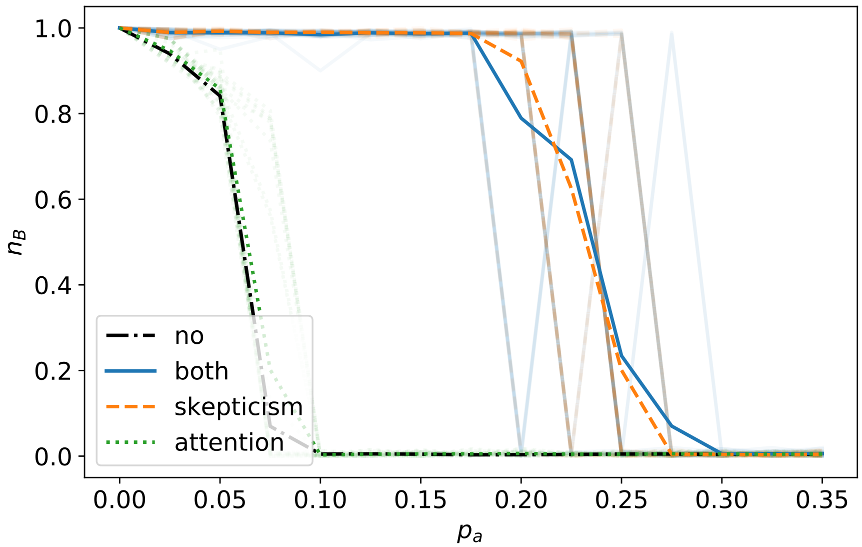

(m) Barabasi-Albert graph with $m = 4$.

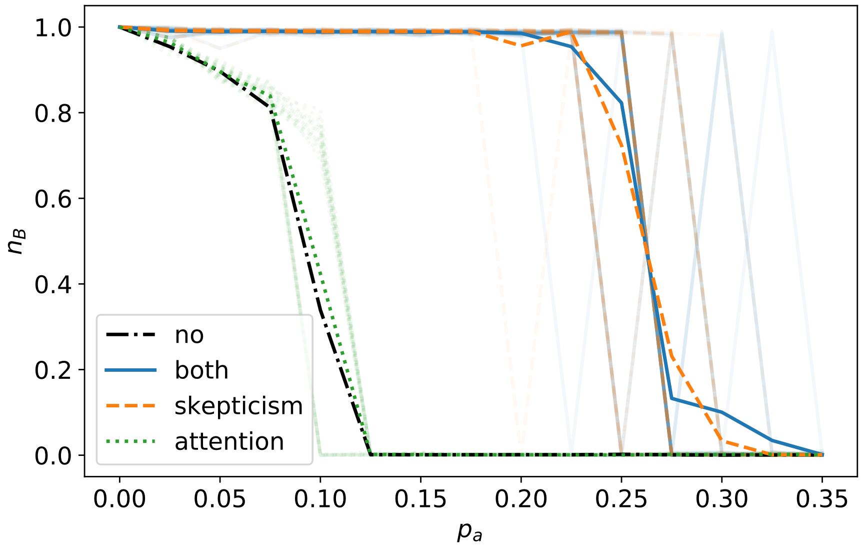

(n) Barabasi-Albert graph with $m = 24$.

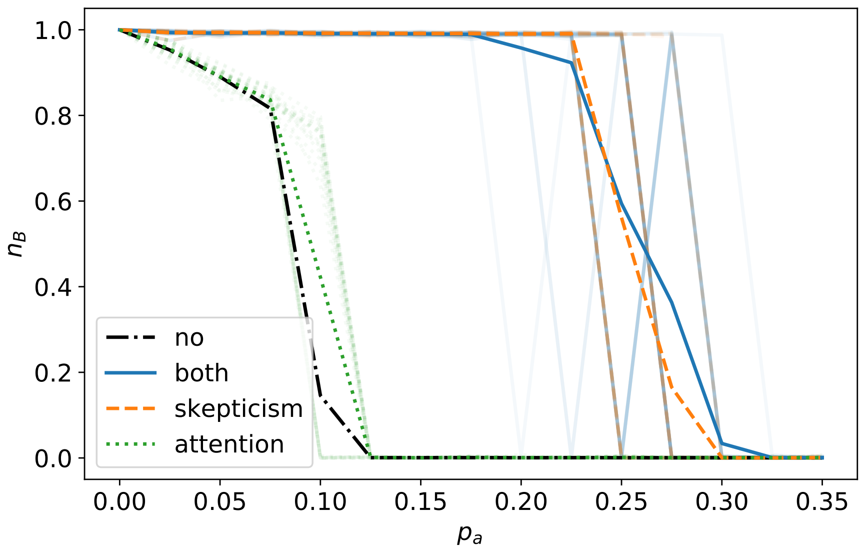

(o) Barabasi-Albert graph with $m = 50$.

4 Counter-Campaign Figures

This section contains all of the results for applying counter-campaigns to various artificial social networks. Each plot corresponds to the graph type listed in the caption. As before, we show the results for no intervention with a black dot-dashed line. We show the effects of a small counter-campaign ($p_b = .05$) with a solid blue line and of a large counter-campaign ($p_b = .15$) with a dashed orange line. Each opaque line shows the average of 10 simulations, and transparent lines show the results for individual runs.

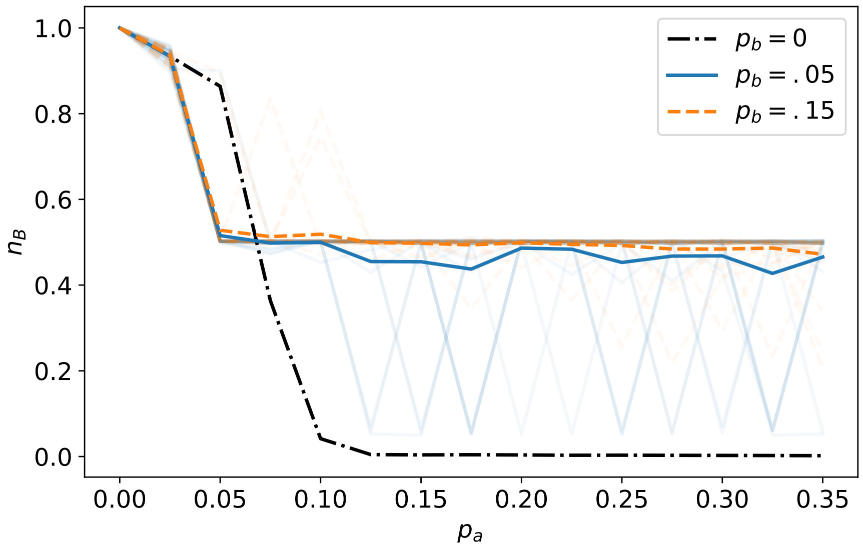

(a) Barbell graph

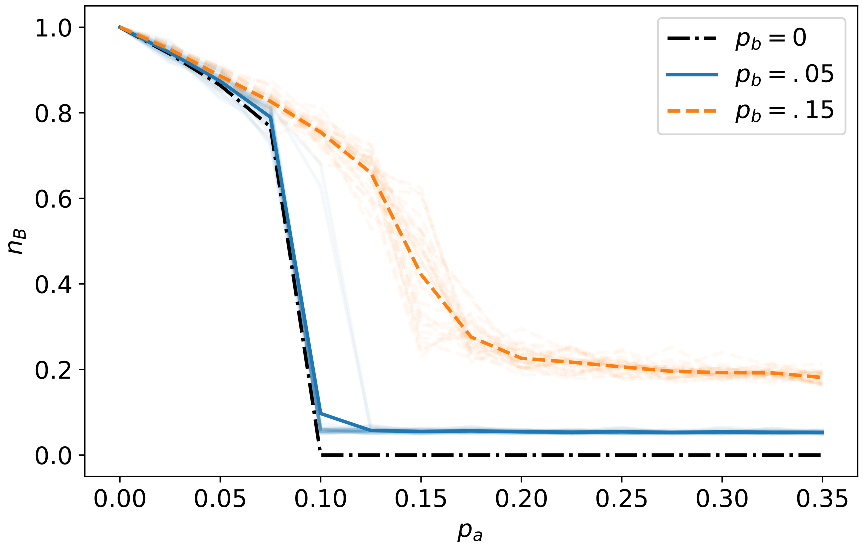

(b) Complete graph

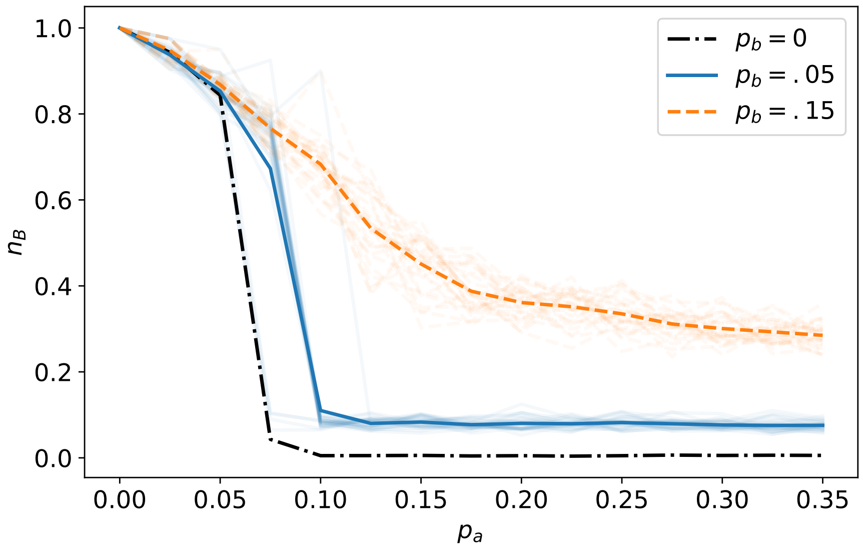

(c) Erdos-Renyi graph with $p = .02$

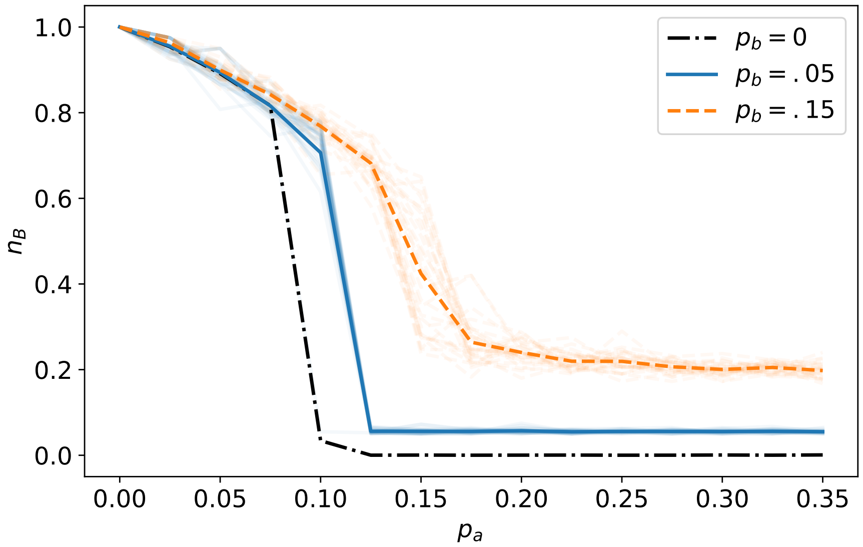

(d) Erdos-Renyi graph with $p = .12$

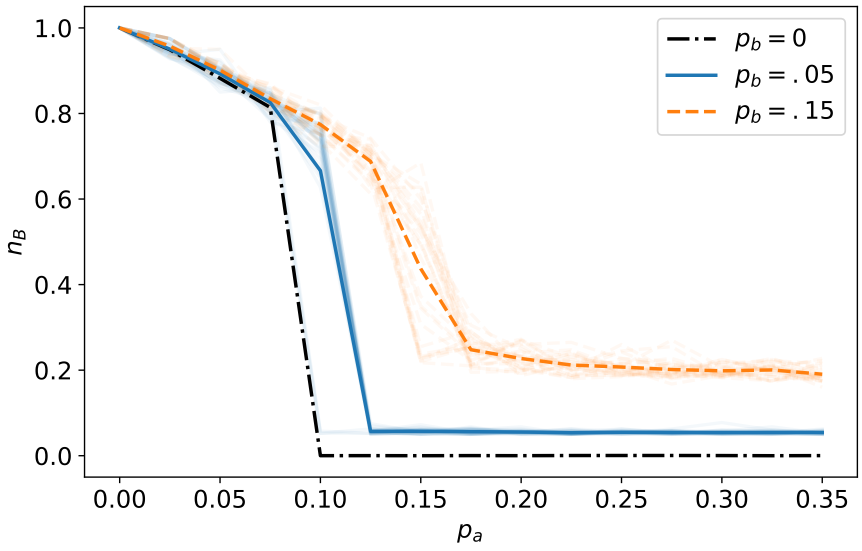

(e) Erdos-Renyi graph with $p = .25$

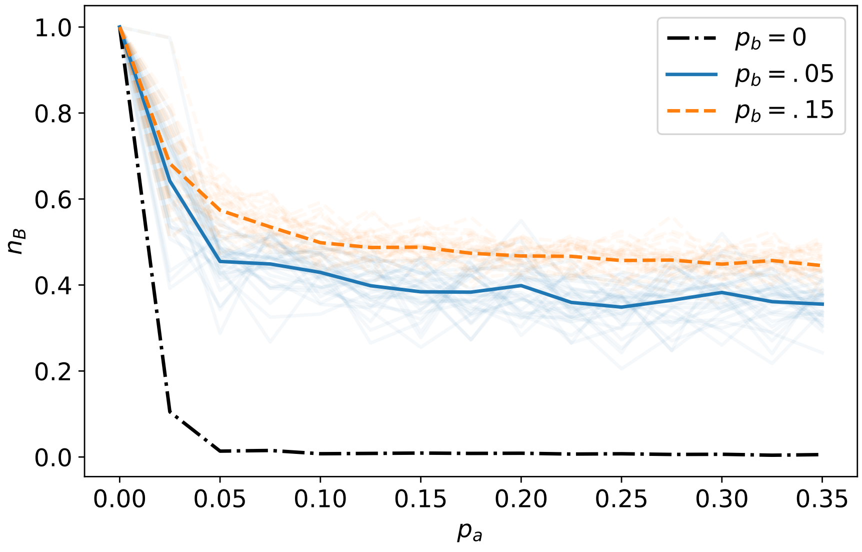

(f) Grid graph

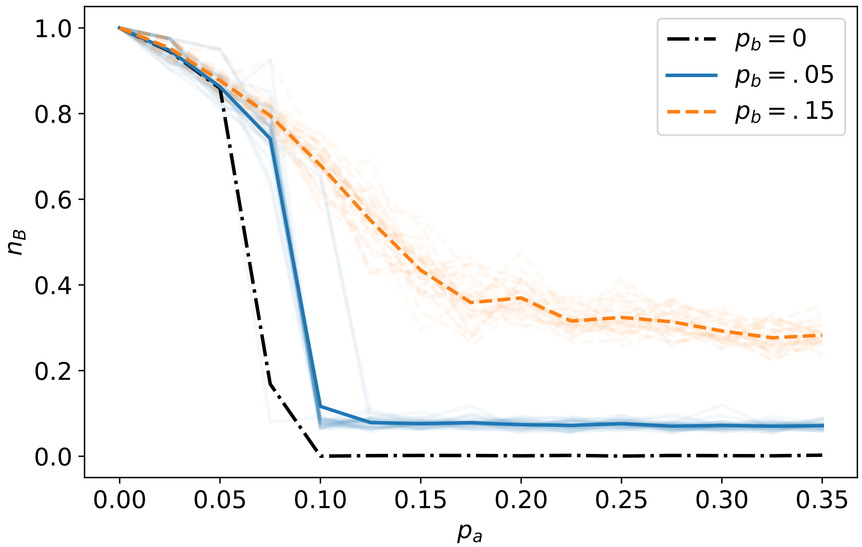

(g) Watts-Strogatz random graph with $k = 8$ and $p = 1$.

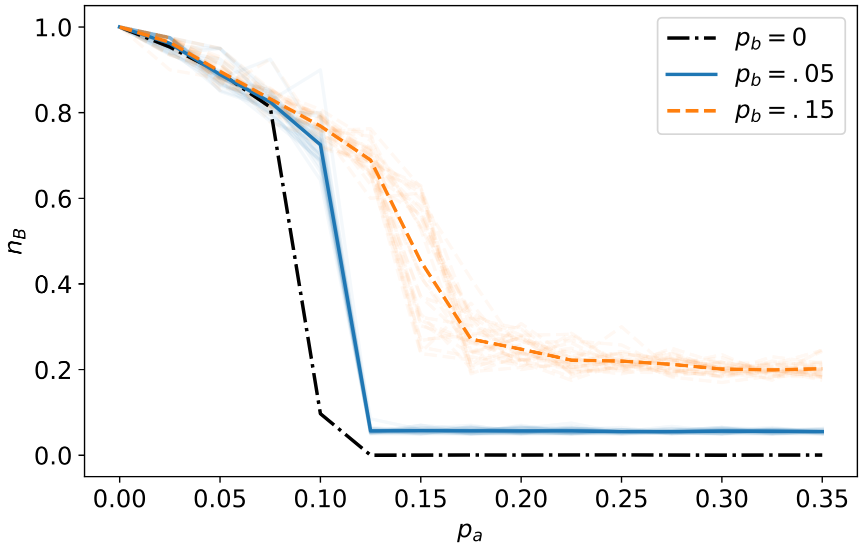

(h) Watts-Strogatz random graph with $k = 48$ and $p = 1$.

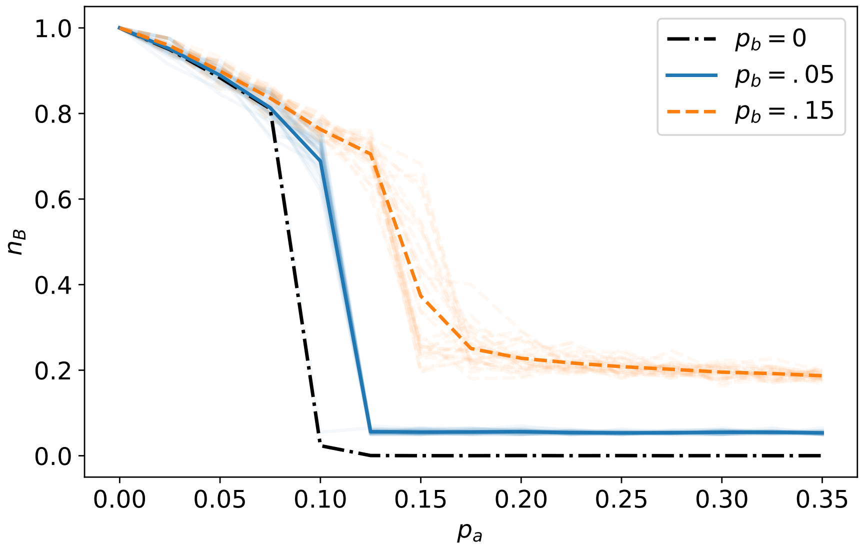

(i) WSR25

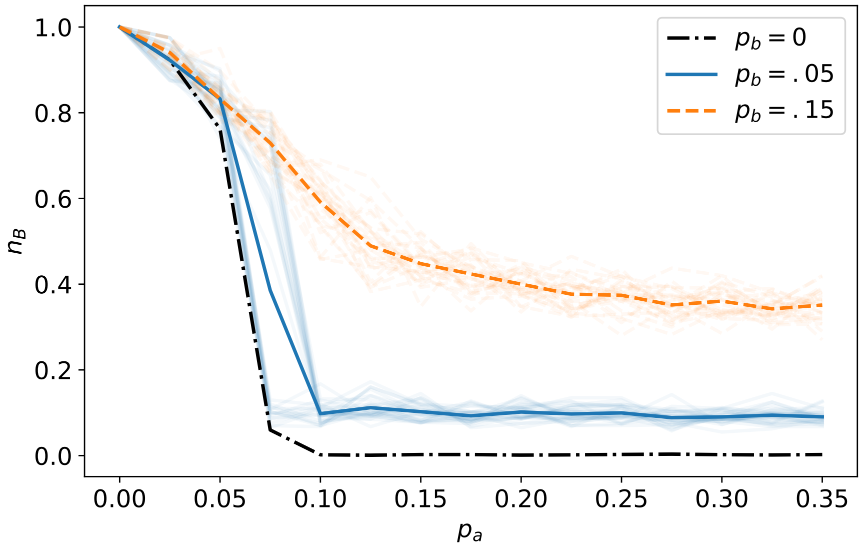

(j) Watts-Strogatz small world graph with $k = 8$ and $p = .5$.

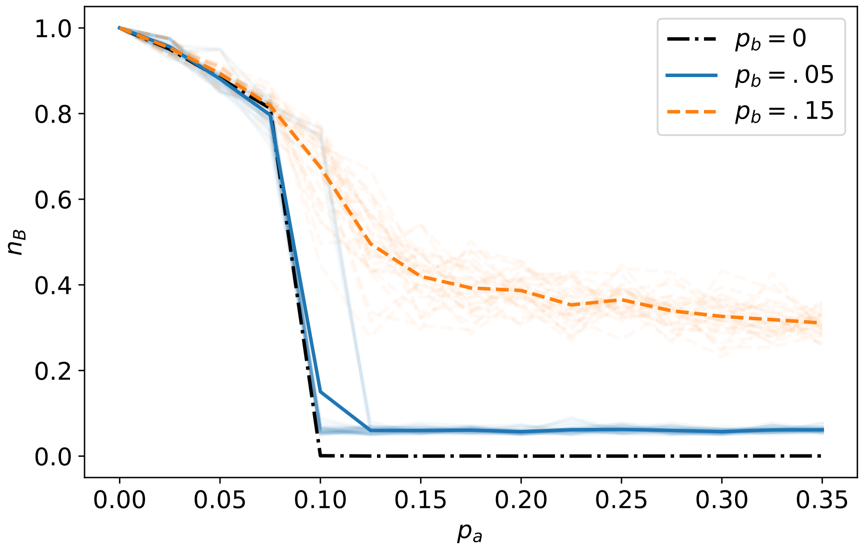

(k) Watts-Strogatz small world graph with $k = 48$ and $p = .5$.

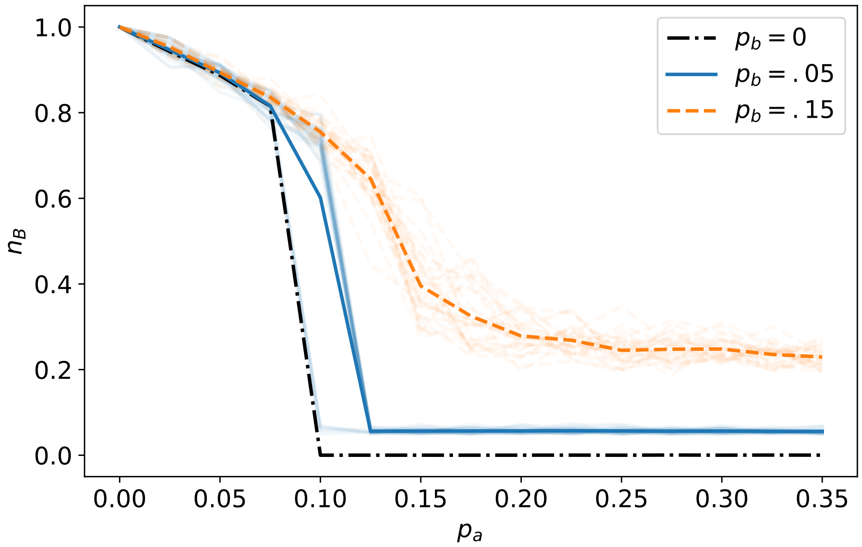

(l) Watts-Strogatz small world graph with $k = 100$ and $p = .5$.

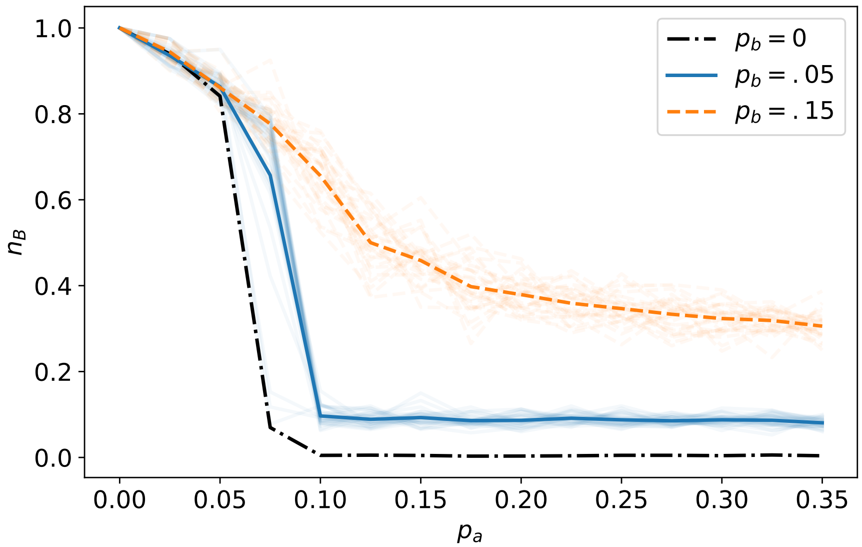

(m) Barabasi-Albert graph with $m = 4$.

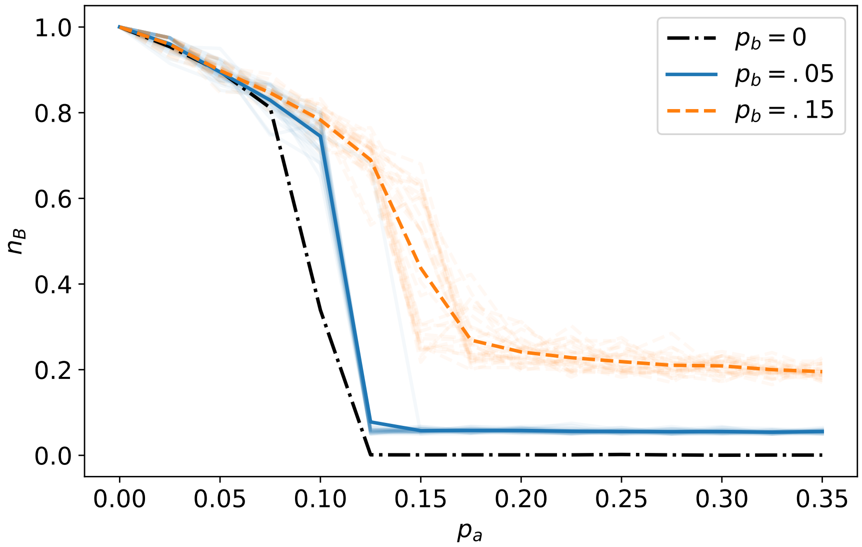

(n) Barabasi-Albert graph with $m = 24$.

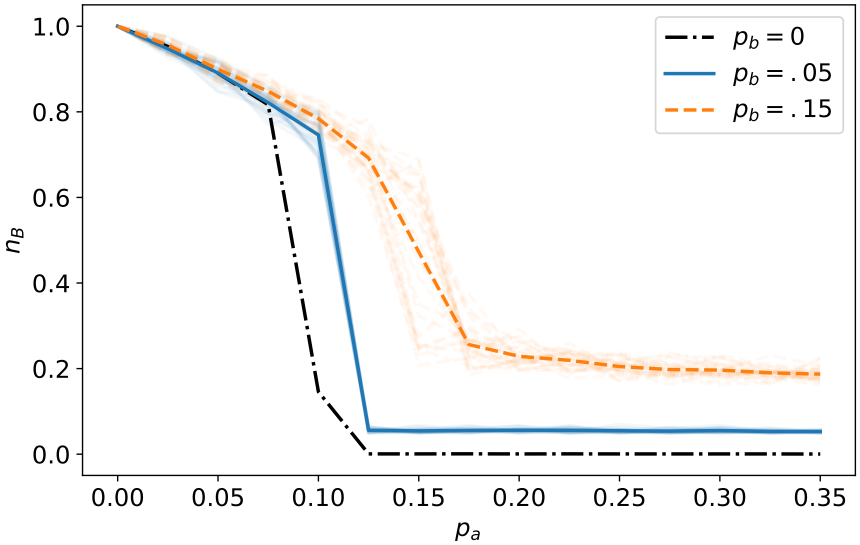

(o) Barabasi-Albert graph with $m = 50$.

References

1. DeGroot, M. H. Reaching a consensus. *J. Am. Stat. association* **69**, 118–121 (1974).

2. Golub, B. & Jackson, M. O. Naive learning in social networks and the wisdom of crowds. *Am. Econ. Journal: Microeconomics* **2**, 112–149 (2010).
